# The amyloid precursor family of proteins in excitatory neurons are essential for regulating cortico-hippocampal circuit dynamics *in vivo*

**DOI:** 10.1016/j.celrep.2025.115801

**Published:** 2025-06-11

**Authors:** Samuel S. Harris, Rikesh M. Rajani, Jana Zünkler, Robert Ellingford, Mengke Yang, James M. Rowland, Alexander Schmidt, Byung Il Lee, Marten Kehring, Mariam Hellmuth, Francesca Kar Wey Lam, Dominique Fässler, Susanne Erdinger, David P. Wolfer, Carlo Sala Frigerio, Fred Wolf, Bradley T. Hyman, Ulrike C. Müller, Marc Aurel Busche

**Affiliations:** 1 UK Dementia Research Institute at University College London, London, UK; 2 British Heart Foundation - UK Dementia Research Institute Centre for Vascular Dementia Research at The University of Edinburgh, Edinburgh, UK; 3 Göttingen Campus Institute for Dynamics of Biological Networks, Göttingen, Germany; 4 Department of Functional Genomics, Institute of Pharmacy and Molecular Biotechnology, Heidelberg University, Heidelberg, Germany; 5 Institute of Anatomy, University of Zurich, Zurich, Switzerland; 6 Institute of Human Movement Sciences and Sport, ETH Zurich, Zurich, Switzerland; 7 Department of Neurology, Massachusetts General Hospital, Harvard Medical School, Charlestown, MA, USA; 8 Department of Neurodegenerative Diseases, University Hospital of Geriatric Medicine FELIX PLATTER and University of Basel, Basel, Switzerland; 9 Department of Biomedicine, University of Basel, Basel, Switzerland; 10 Lead contact

## Abstract

The amyloid precursor protein (APP) family is ubiquitously expressed in the mammalian brain and implicated in Alzheimer’s disease. APP family proteins participate in synaptic function and their absence impairs cognition. However, how these proteins regulate neural circuits and influence brain-behavior relationships remains unknown. Using *in vivo* two-photon Ca^2+^-imaging and Neuropixels, we show that APP family knockout (KO) in excitatory neocortical and hippocampal neurons suppresses neuronal dynamics across behavioral states, and results in an increased proportion of low-activity and silent neurons. Further, APP family KO leads to a reduction in synapses expressing the requisite N-methyl-D-aspartate receptor (NMDAR) subunit GluN1, with pharmacological enhancement of NMDAR function normalizing aberrant dynamics in low-activity neurons and rectifying behavioral impairments. Suppressing NMDAR function in control mice replicates the functional phenotype observed in APP family KOs. Our findings indicate a physiological role for the APP family in regulating and sustaining spontaneous neuronal activity in cortico-hippocampal circuits *in vivo*.

## In brief

Harris et al. show that absence of the APP family in excitatory neurons is associated with suppression and silencing of corticohippocampal circuits and behavioral deficits that are linked to impaired NMDAR function. These findings indicate an essential role for the APP family in maintaining normal neuronal circuit function *in vivo*.

## Graphical Abstract

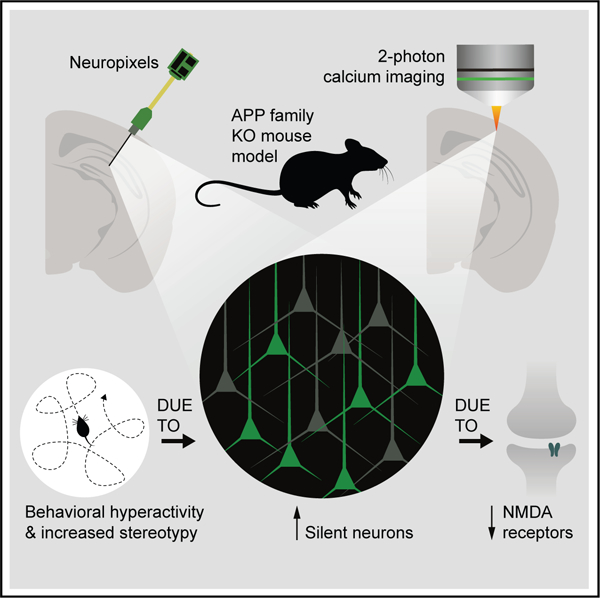

## INTRODUCTION

The amyloid precursor protein (APP), initially described in 1987,^1–3^ is the precursor to the amyloid-beta (Aβ) peptide that deposits in the brain of individuals with Alzheimer’s disease (AD).^4^ In mammals, it has two homologous proteins, APLP1 and APLP2, that possess a similar structure but do not contain the Aβ domain. Emerging evidence implicates the APP family in several other diseases where synaptic and neural circuit alterations are proposed hallmarks, including schizophrenia, autism, and epilepsy.^5–8^ The physiological function of APP proteins in the intact brain has remained enigmatic, but several lines of evidence point to an essential role for this family of proteins in brain function, including their contribution to approximately 1% of all synaptic proteins,^9^ expression across multiple brain cell types, evolutionary conservation, and functional overlap and structural homology among family members.^1,10,11^ Their functional importance is further supported by reports of multi-domain cognitive deficits in double or triple knockouts (KOs) of the APP family,^12–17^ and which are suggestive of a key role in modulating neural circuits that shape cognition and behavior. Accompanying *in vitro* slice preparations have shown altered synaptic plasticity and neuronal excitability, primarily in the hippocampus, in the absence of the APP family.^12,13,15,17^ However, these findings do not fully account for the extensive deficits in behavior and cognition, which point to a wider impairment of neuronal function beyond the hippocampus. Indeed, RNA sequencing (RNA-seq) analyses in a triple KO of the APP family revealed prominent gene expression changes not only in the hippocampus but also in the neocortex,^18^ although the consequences of these findings for the function of cortico-hippocampal circuits have remained unclear.

A fundamental property of cortico-hippocampal circuits is the intrinsic manifestation of structured patterns of neuronal activity. Such spontaneous activity, which relies on intact synaptic transmission,^19^ has been shown to integrate and represent sensory and multidimensional behavioral information,^20^ and subserves computational functions related to memory, learning, and decision-making.^21,22^ We hypothesized that APP family KO adversely impacts spontaneous neuronal activity in cortex and hippocampus, which reflects an underlying cellular/synaptic impairment that could in turn mechanistically underpin the wide-ranging behavioral and cognitive deficits previously observed in these animals.^12^

To test this, we combined several techniques to gain a multiscale and multi-regional understanding of the physiological role of the APP family in the intact living brain, which has remained largely unknown to date. First, we took advantage of a recently established conditional triple KO (cTKO) mouse model with deletion of the entire APP family in excitatory forebrain neurons.^12^ This model surmounts the lethality of double and triple germline KOs, while circumventing functional overlap within the APP family.^14,23^ Second, we employed two-photon Ca^2+^-imaging and multi-regional Neuropixels recordings to monitor spontaneous neuronal and circuit activity with single-cell resolution across behavioral states in cortical regions (retrosplenial and visual cortex) and hippocampal CA1. We selected these regions as APP family proteins are highly expressed in these areas under normal conditions, they corresponded to anatomical locations where the APP family was absent in excitatory neurons in cTKO mice, and include brain areas tightly linked to vulnerability to AD pathology.^24,25^ We observed that KO of the APP family within excitatory forebrain neurons resulted in a suppression of ongoing activity during awake rest and locomotion states as well as sleep-related slow-wave activity (SWA). We found that a large proportion of neurons in cortical regions were highly hypoactive and functionally inactive in cTKO mice, suggesting that the influence of the APP family extends beyond the hippocampus. A fundamental property of neuronal circuits *in vivo* is the adjustment of activity levels across behavioral states, such as changes in neuronal activity during locomotion,^26^ a capability we found to be impaired in cTKO mice, particularly with respect to low-activity neurons. Computational modeling revealed that deficits in N-methyl D-aspartate receptor (NMDAR) function might underpin these neuronal impairments. Experimental validation showed that NMDARs are indeed required for ongoing neuronal activity, as suppressing NMDAR function resulted in a reduction in neuronal activity levels and an increase in the number of inactive neurons. Accordingly, we found that the obligatory GluN1 NMDAR subunit was reduced in cTKO mice, and that partial NMDAR agonism selectively enhanced neuronal dynamics in low activity neurons of cTKO mice and ameliorated behavioral impairments. Together, these results demonstrate that the presence of the APP family in excitatory neurons is essential for maintaining spontaneous neuronal activity *in vivo*, and that their interaction with NMDARs is a critical determinant of this function.

## RESULTS

### Loss of the APP family is associated with suppressed neuronal dynamics during awake resting and locomotion states

Given the pan-cellular expression of the APP family, exemplified in our re-analysis of publicly available mouse and human transcriptomic data^27^ (Figure S1), and to circumvent the lethality of germline TKO mice, we generated a cTKO model (NexCre-cTKO, referred to here as cTKO) of the APP family in excitatory forebrain neurons by crossing APP^flox/flox^/APLP2^flox/flox^/APLP1^−/−^ mice with NexCre mice expressing Cre prenatally at approximately E11.5, and permanently thereafter, in post-mitotic neuronal precursor cells of the neocortex and hippocampus (as recently described in Steubler et al.^12^; see also previous examinations of NexCre conditional double KO mice in which a selective deletion of APP in excitatory neurons of both cortex and hippocampus was confirmed^15,16^). Note that while APP and APLP2 alleles were floxed and deleted in a NexCre-specific pattern,^28^ APLP1 was constitutively inactivated in the germline. This early embryonic time point of Cre expression is particularly relevant, since APP family expression increases from E14 onward and reaches a peak during synaptogenesis.^29^ Consequently, our model represents an advance over a recently published alternative,^13^ in which APP family proteins were postnatally deleted after this critical early development period (at day ∼18), and showed incomplete gene inactivation with residual APLP1 expression at approximately one-half that of wild-type (WT) mice. Age-matched littermates lacking APLP1 (APP^flox/flox^/APLP2^flox/flox^/APLP1^−/−^), which exhibit no functional or cognitive deficits and resemble WT mice,^30^ were used for control comparisons.

**Figure 1.**
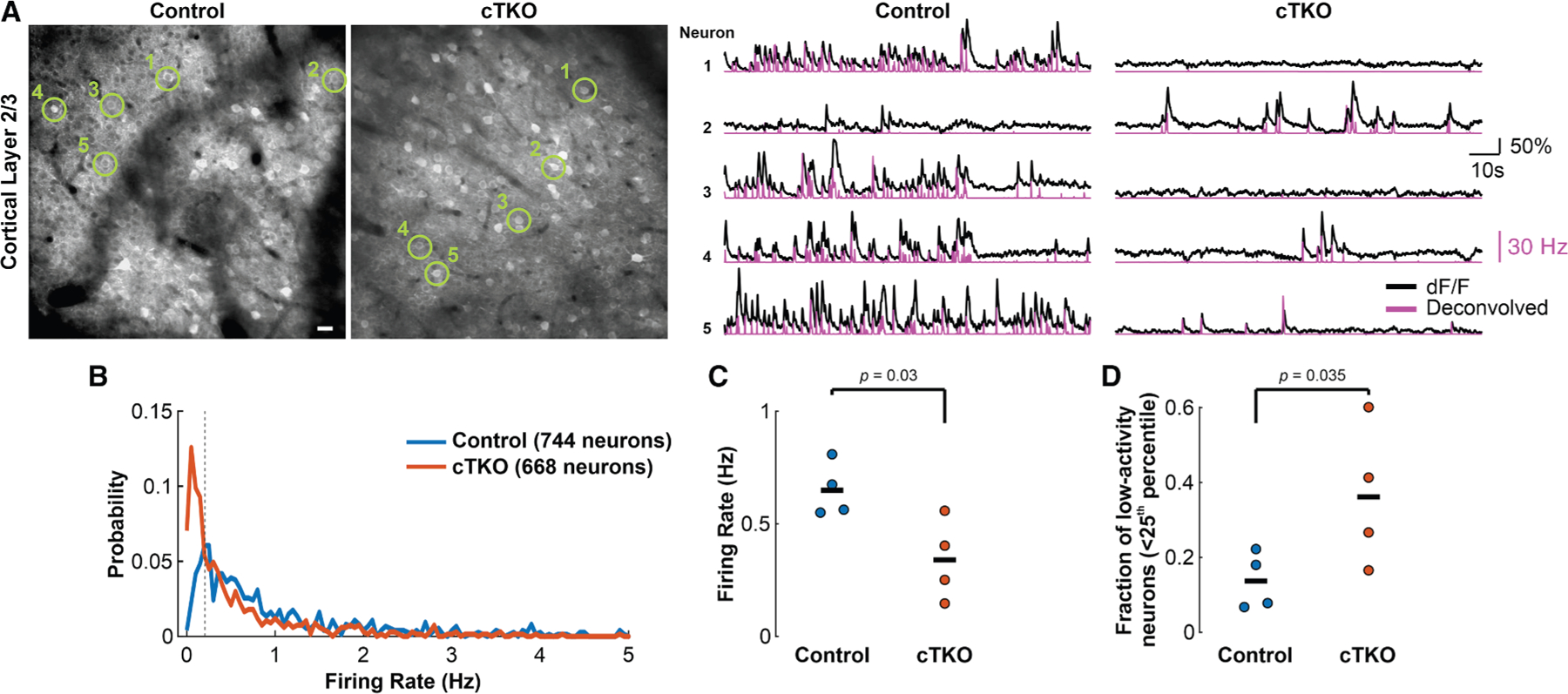
Cortical neuron firing during awake rest is suppressed in the absence of the APP family (A) Example *in vivo* two-photon fluorescence images of GCaMP8s-expressing layer 2/3 neurons in retrosplenial cortex of a control (left) and cTKO (right) mouse during awake rest. Right, spontaneous Ca^2+^ activity (dF/F) and deconvolved traces from five example neurons circled on the left for each genotype. Scale bar, 25 μm. (B) Probability distribution plot of single-neuron firing rates in layers 2/3 across genotypes (744 and 668 neurons from *N* = 4 control and 4 cTKO mice, respectively) highlighting a shift toward hypoactive firing rates in the cTKO condition. (C) Quantification of neuronal firing rates at the animal level revealing significantly reduced firing in cTKO mice relative to controls, t test, t(6) = −2.9, *p* = 0.03, each data point represents an individual animal, *N* = 4 mice per genotype. (D) Fraction of low-activity neurons, indicating increased prevalence of these neurons in cTKO mice versus controls, one-tailed t test, t(6) = 2.2, *p* = 0.035, each data point represents an individual animal, *N* = 4 mice per genotype. See also Figure S2 and Table S1.

To assess how the selective absence of the APP family in excitatory neurons affects neuronal dynamics, we performed two-photon Ca^2+^-imaging of retrosplenial cortex in awake head-fixed mice supported by a 3D-printed tube (in darkness and in the absence of external stimuli). We expressed the genetically encoded calcium indicator jGCaMP8s^31^ (under the synapsin promoter) in layer 2/3 cortical neurons, and estimated the average firing rate for each neuron by denoising and deconvolving the ΔF/F traces, then averaging across each frame, as previously described^32,33^ (Figure 1A). These experiments revealed that cTKO mice exhibited significantly suppressed awake resting neuronal firing rates compared with controls, and an increase in the proportion of low-activity neurons (Figures 1B–1D). Analogous effects were also observed when estimating Ca^2+^-transient frequency from the deconvolved trace in each cell (Figure S2A). Since the hypoactivity phenotype in cTKO mice might be explained and confounded by reduced movement relative to controls,^34^ we also computed the percentage of time each mouse spent moving during the recording. cTKO mice were observed to move more than controls during recordings, albeit not significantly (Figure S2B), and, importantly, the significant reduction in firing rate we previously observed remained even after inclusion of the movement metric as a fixed effect in a linear mixed effects (LME) model that accounted for inter-subject variability (Table S1).

**Figure 2.**
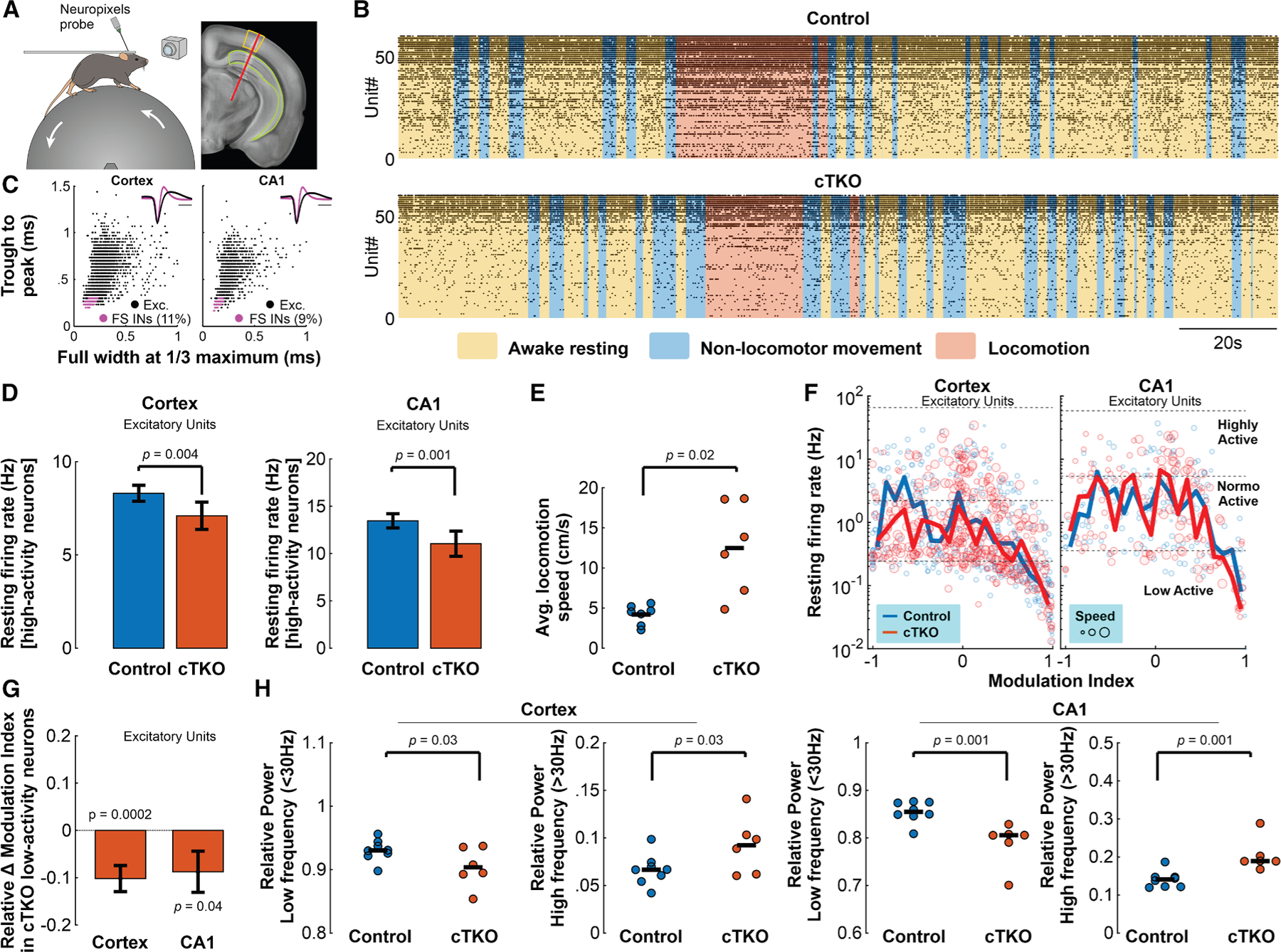
Absence of the APP family attenuates neuronal dynamics in cortex and CA1 across awake behavioral states at the single neuron and population levels (A) Left, schematic of Neuropixels implantation in awake animals alongside behavioral monitoring. Right, compound image of mesoSPIM light-sheet data, comprising DiI-labeled Neuropixels probe track (red) and brain structure, overlaid with spatially registered Allen CCF v3 atlas image and regions of interest (visual cortex and hippocampus CA1). (B) Example raster plots from all units in regions of interest during awake behavior in a control (top) and cTKO (bottom) mouse (sorted by average firing rate). (C) Units were classified into putative excitatory and inhibitory fast-spiking interneurons (FS INs) for each region of interest using Gaussian mixture modeling of spike waveform properties. Scale bar in insets, 0.5 ms. (D) Resting firing rates of excitatory high-activity neurons were reduced in cTKO mice versus controls in both cortex and CA1 (*N* = 8 controls, 6 cTKO). Data represent LME-model-derived estimates with standard errors, accounting for both fixed and random effects. Cortex, Control (from *N* = 8 mice): 43.4 ± 6.8 neurons/session/animal; cTKO (from *N* = 6 mice): 56.9 ± 14.1 neurons/session/animal. CA1, control (from *N* = 8 mice): 18 ± 3.5 neurons/session/animal; cTKO (from *N* = 6 mice): 23.3 ± 5.1 neurons/session/animal. (E) Significant difference in average locomotion speed between control and cTKO mice, Welch’s t test, t(5.3) = −3.44, *p* = 0.02, *N* = 7 control, *N* = 6 cTKO, one control mouse immobile during recordings. (F) Relationship between resting firing rates and firing rate modulation by locomotion (modulation index, MI), indicating that low-firing excitatory neurons in cortex and CA1 are particularly predisposed to relatively large changes in firing during locomotion. Each data-point represents an individual excitatory neuron from one example experimental session across animals (Cortex: *n* = 402 control neurons from 7 animals, *n* = 384 cTKO neurons from 6 animals, one control mouse immobile during recordings; CA1: *n* = 169 control neurons from 7 animals, *n* = 158 cTKO neurons from 6 animals) with the diameter of each marker indicating normalized locomotion speed. Horizontal lines indicate threshold for each resting state firing category (Low active, Normoactive and Highly active) and brain region. (G) The relationship between locomotion speed and neuronal firing rate modulation by locomotion (modulation index, MI) was significantly and consistently reduced in excitatory low-activity neurons of cTKO mice versus controls in both cortex and CA1. Data represent LME model estimates with standard errors, accounting for both fixed and random effects. Cortex, Control (from *N* = 7 mice, one animal immobile during recordings): 45.8 ± 7.4 neurons/session/animal; cTKO (from *N* = 6 mice): 57.6 ± 13.8 neurons/session/animal. CA1, Control (from *N* = 7 mice, one animal immobile during recordings): 19.1 ± 3.9 neurons/session/animal; cTKO (from *N* = 6 mice): 21.8 ± 5.3 neurons/session/animal. (H) Relative resting state LFP power in low-frequency (<30 Hz) and high-frequency (>30 Hz) bands were significantly decreased and increased, respectively, in cortex and CA1 of cTKO mice versus controls (each data point represents an individual animal). (D,G,H) *p*-values were obtained from LME models and provided as insets with statistical details provided in Tables S2–S4. See also Figure S2.

To examine whether these changes in neuronal dynamics extended to brain regions beyond the retrosplenial cortex, as well as active behavioral states, and assess action potential activity more directly, we conducted Neuropixels recordings traversing visual cortex and hippocampus CA1 in awake head-fixed adult cTKOs and littermate controls during which mice were free to rest or run on a rolling Styrofoam wheel, while locomotor movement and pupil size were monitored using a rotary encoder and camera (Figures 2A and S2C). Spontaneous neuronal activity recordings across behavioral states were again conducted in darkness and in the absence of external stimuli (Figure 2B). After classifying spike-sorted units into putative excitatory and fast-spiking inhibitory neuron types based on spike waveform properties (Figure 2C), we first assessed single-cell firing differences between genotypes during the awake resting state using LME models accounting for nested random effects of individual animals and recording sessions. Subsequently, after confirming that arousal levels (indexed by pupil diameter) during the resting state did not differ significantly between genotypes (Figure S2D), we categorized neurons into three activity classes based on the upper and lower quartiles of the firing rate distribution within each brain region and neuronal type across genotypes, to examine the effects of APP KO on these functional subpopulations. Neurons were classified as low activity (<25th percentile), normoactive (25th–75th percentile, reference level), or high activity (>75th percentile). We observed that the awake resting state firing rates of high-activity excitatory neurons in both brain regions were selectively reduced in cTKO animals, suggesting a deficit in the regulation or maintenance of elevated spontaneous activity in such neurons (Figure 2D; Table S2). A similar effect was also observed in putative inhibitory interneurons in the cortex (which are not affected by the KO), but not those of hippocampus CA1 (Table S2).

The cTKO animals locomoted at significantly faster speeds than their control counterparts, albeit not for significantly different fractions of recording time, consistent with a previous report^12^ (Figures 2E and S2E). Since locomotion modulates neuronal activity in visual cortex,^34^ we examined neuronal firing rates during such behaviors, and their relationship to resting state firing levels. The relationship between activity rates during awake resting and locomotion states was approximately linear and positive for excitatory neurons which were normally and highly active during rest, but inversely correlated for low-activity excitatory neurons, in both cortex and CA1 (Figure S2F). LME modeling indicated that this relationship in normo-active and highly active excitatory neurons was altered in cTKO mice versus controls in both brain regions, and was not better described by the addition of locomotion speed as a main effect in the model (Figure S2G). To examine these differences in greater detail, we calculated the firing rate modulation index $\left[\text{MI}=\left(\text{LocFR}-\text{RestFR}\right)\text{/}\left(\text{LocFR}+\text{RestFR}\right)\right]$ for each neuron, which normalizes for absolute firing rate differences between locomotion (LocFR) and resting states (RestFR).^26^ Comparison of resting firing rates and the MI revealed that the low-activity excitatory neurons were selectively predisposed to large relative increases in firing, in both cortex and CA1 (Figure 2F). This is notable, as low-activity neurons have been implicated to support encoding and integration of novel information during behavior.^35,36^ We subsequently leveraged an LME model to quantify the relationship between resting firing rate category, locomotion speed and genotype on MI (incorporating speed as part of a three-way interaction statistically improved model accuracy over a model containing only a two-way interaction between the other variables) (Table S3). This analysis revealed a consistent impairment of resting low-activity excitatory neurons across brain regions to dynamically adapt their firing patterns according to locomotion speed in cTKO mice, indicating a vulnerability of these neurons to a loss of the APP family (Figures 2G; Table S3). Lastly, we examined population-level local field potential (LFP) activity in the cortex and CA1 during awake resting states with particular interest on aberrant neural synchrony, which might allude to an altered ability of cTKO brains to support low- and high-frequency oscillatory rhythms. Indeed, we observed a significant reduction in relative LFP power at low-frequencies (<30 Hz) alongside a corresponding increase in high-frequency power (>30 Hz^37^) across both brain regions in cTKO mice, relative to controls (Figures 2H and S2H; Table S4). Taken together, our data point to an attenuation of spontaneous neuronal dynamics in cTKO mice during awake states, with an overabundance and impairment of low-activity neuron populations, the latter leading to inadequate scaling of resting-state neuronal activity to meet behavioral demands.

### Absence of the APP family promotes marked neuronal silencing during SWA

**Figure 3.**
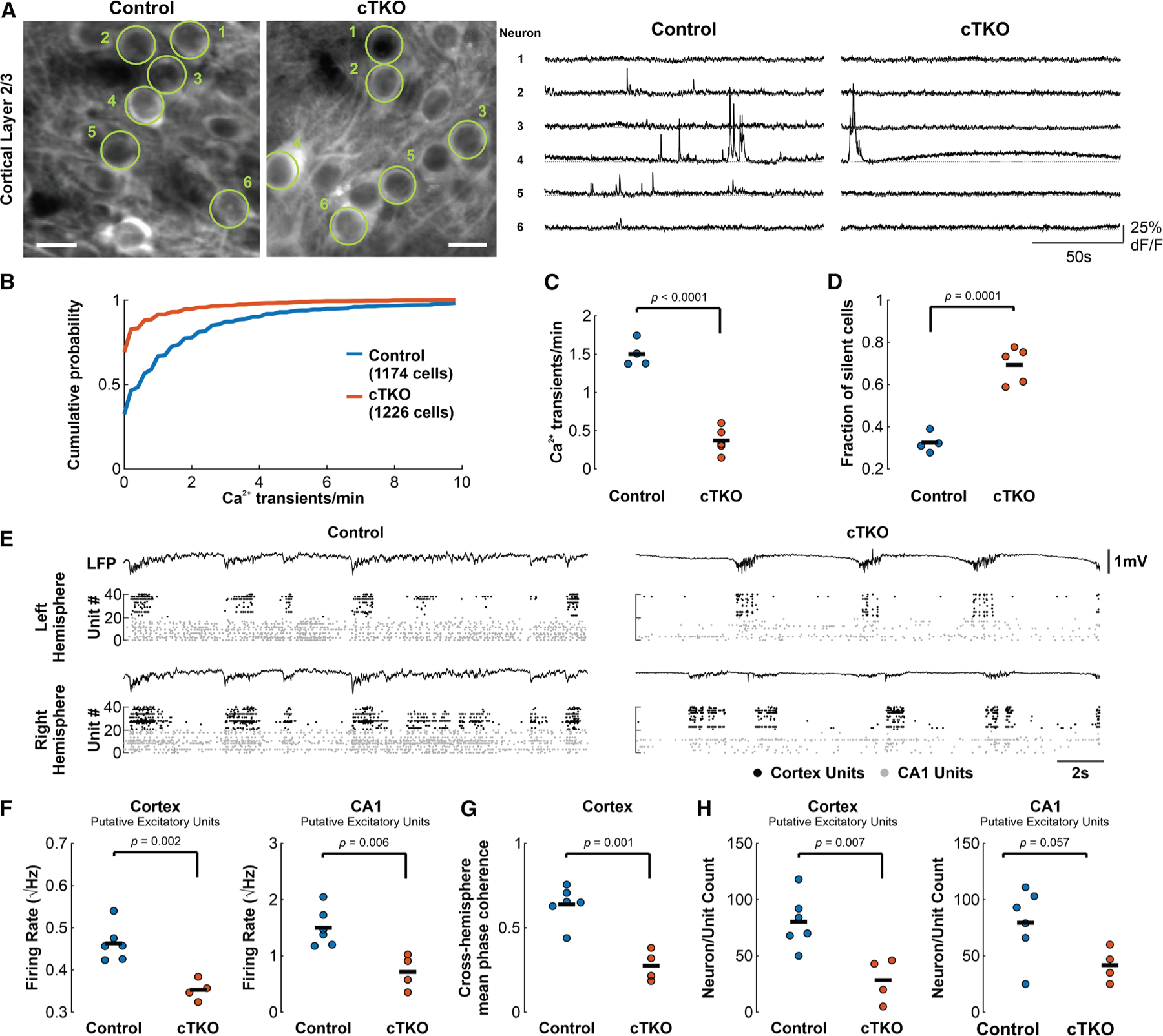
Neuronal firing impairments in cTKO mice are exacerbated during SWA with profound suppression and silencing of neurons (A) Left, example *in vivo* two-photon fluorescence images of GCaMP6f-expressing layer 2/3 neurons in visual cortex of a control (left) and cTKO (right) mouse during SWA. (Right) Spontaneous Ca^2+^-activity (dF/F) from six example neurons circled on the left for each genotype. Scale bars, 10 μm. (B) Cumulative distribution plot displaying neuronal Ca^2+^-transient rates of individual neurons in layers 2/3 across genotypes (neurons from *N* = 4 control and *N* = 5 cTKO mice) and indicating a shift toward hypoactivity in the cTKO condition. (C) Quantification of neuronal Ca^2+^-transient rates at the animal level, showing neuronal hypoactivity in cTKO mice versus controls during SWA, *N* = 4 control, *N* = 5 cTKO, t test, t(7) = 9.7, *p* < 0.0001, each data point represents an individual animal. (D) Significant increase in the fraction of functionally silent neurons in cTKO mice compared with controls, *N* = 4 control, *N* = 5 cTKO, t test, t(7) = −7.6, *p* = 0.0001, each datapoint represents an individual animal. (E) Example raster plots of Neuropixels recordings from 40 randomly selected cortical (black) and CA1 (gray) units/neurons and cortical LFP traces in both hemispheres during SWA in a control mouse (left) and cTKO mouse (right). (F) Neuronal firing in excitatory neurons was significantly suppressed in cTKO mice versus controls in both cortex and CA1, each data-point represents an individual animal; *N* = 6 control, *N* = 4 cTKO; Cortex, t test, t(8) = 4.6, *p* = 0.002; CA1, t test, t(8) = 3.7, *p* = 0.006. (G) Significant attenuation of cross-hemispheric cortical mean phase coherence (MPC) in cTKO mice compared with controls (see also example in E, right panel; each data point represents an individual animal, *N* = 6 control, *N* = 4 cTKO, t test, t(8) = −5.51, *p* = 0.001). (H) The number of excitatory units/neurons identified across both hemispheres was significantly reduced in cTKO mice in cortex relative to controls, with a borderline reduction in CA1, each data point represents an individual animal, *N* = 6 control, *N* = 4 cTKO; Cortex: t test, t(8) = 3.6, *p* = 0.007; CA1: t test, t(8) = 2.2, *p* = 0.057. See also Figure S3.

To further understand the deficits in low-frequency behavior in neuronal populations, we employed low doses of isoflurane to reliably induce robust cortical slow-wave oscillations.^38,39^ These oscillations reflect alternations between periods of neuronal spiking (UP states) and periods of silence (DOWN states), and are critically involved in the consolidation of recently learned memories^40^ and synaptic homeostasis^41^ during non-REM sleep. We performed two-photon Ca^2+^-imaging of GCaMP6f-expressing layer 2/3 visual cortex neurons (again under the control of a synapsin promoter) *in vivo* to provide further insight into their dynamics (Figure 3A). These experiments indicated that cortical neurons in cTKO mice were associated with a suppression in spontaneous activity relative to controls during SWA, and also revealed a pronounced increase in the proportion of functionally silent or inactive neurons, with approximately 70% of all recorded neurons in cTKO mice failing to spontaneously generate Ca^2+^ transients (Figures 3B–3D). The suppression of neuronal activity and the marked increase in silent/hypoactive neurons *in vivo* during SWA are consistent with our awake two-photon Ca^2+^-imaging findings, and suggests a profound and wide-ranging disruption of normal cortical function in cTKO mice.

We also performed bilateral neuropixel recordings of the visual cortex and hippocampus CA1 during SWA (Figure 3E), and observed a marked reduction of cortical and CA1 neuronal activity, primarily in putative excitatory neuron populations, in cTKO mice versus controls (Figures 3F and S3A). Furthermore, to examine the individual contribution of the other APP family members, we conducted similar experiments in constitutive APP and APLP2 single KO mice (with gene deletion in all cell-types) as well as their WT counterparts. We found no differences in excitatory neuron mean firing rates in cortical and CA1 regions in individual KOs versus WTs (Figure S3B), suggesting that the deletion of single APP family members is not sufficient to induce attenuation in neuronal dynamics, likely due to a functional overlap and compensation between family members.

Similarly to our awake findings, we observed a marked reduction in within-hemisphere low-frequency (slow-wave 0.1–1 Hz) oscillatory power, and a contrasting increase in high-frequency power (gamma band 30–90 Hz), in cTKO mice relative to controls in cortex, as well as a prominent de-coherence in population cortical activity between hemispheres during SWA (Figures 3G and S3C). Although the fraction of time spent in a cortical UP state did not differ between genotypes, cortical UP states were significantly shorter lived in cTKO mice versus controls (Figure S3C). A fundamental property of slow waves is that they propagate as traveling waves across the cortex, thereby promoting the coherence of neuronal activity over large cortical territories.^39,42^ This facilitates the transfer of information between distant regions, including the hippocampus, and organizes neurons into transient functional assemblies to support synaptic plasticity and cognitive processes.^43^ Thus, to examine the spatiotemporal propagation of slow waves in greater detail, we performed fast widefield (one-photon) Ca^2+^-imaging of the GCaMP6f-expressing cortical surface with high spatial resolution and coverage. These experiments not only pointed toward deficits in cortical slow wave synchronization between hemispheres in cTKO mice relative to controls but also indicated marked decoherence of this rhythm within the same hemisphere, indicative of widespread spatial impairments in slow-wave dynamics and consistent with our LFP results (Figure S3D). Since cTKO mice often show dysgenesis and agenesis of the corpus callosum,^12^ likely underpinning our observation of reduced cross-hemisphere cortical coherence (Figure 3G), we additionally examined whether such structural abnormalities could impact the above results. To this end, we conducted parallel experiments in BTBR T^+^ Itpr^tf^/J (BTBR) mice, which are characterized by a complete absence of the corpus callosum.^44^ These experiments indicated a significant reduction in cross-hemispheric cortical coherence in BTBR mice relative to controls, but, critically, without a reduction in cortical neuronal firing (Figure S3E). These results suggest that, while abnormalities in the corpus callosum may contribute to the reduction in cross-hemispheric coherence in cTKO mice, they do not appear to explain the marked deficits in within-hemisphere cortical neuronal dynamics, including the impaired slow-wave propagation and reduced single-neuron activity observed in cTKO mice.

Interestingly, we found that the number of functionally identified putative excitatory neurons (i.e., those in which the APP family was specifically deleted), in the cortex of cTKO mice, was markedly and significantly reduced compared with controls, with a borderline significant reduction in CA1 (Figure 3H). Importantly, this appeared to reflect a reduction in the number of functionally active neurons rather than a smaller underlying population of excitatory cells, consistent with normal pyramidal cell numbers in the CA1 region and normal cortical volumes in cTKO mice.^12^ Since Neuropixels recordings are fundamentally blind to functionally silent and highly hypoactive neurons, we conclude that the reduction in detected cells reflects an increased population of silent/hypoactive neurons in cTKO mice (as observed in our two-photon imaging data).

### Computational and pharmacological evidence implicates NMDAR hypofunction as a mechanistic driver of functional impairments in cTKO mice

We next sought to identify possible mechanistic underpinnings of the observed functional impairments in cTKO mice. Guided by our data indicating attenuated spontaneous neural dynamics, we hypothesized an impairment in glutamatergic synaptic transmission, and specifically a compromise in NMDAR-associated function, since our observations of reduced low-frequency LFP power have also been implicated as electrophysiological signatures of NMDAR hypofunction.^45,46^

**Figure 4.**
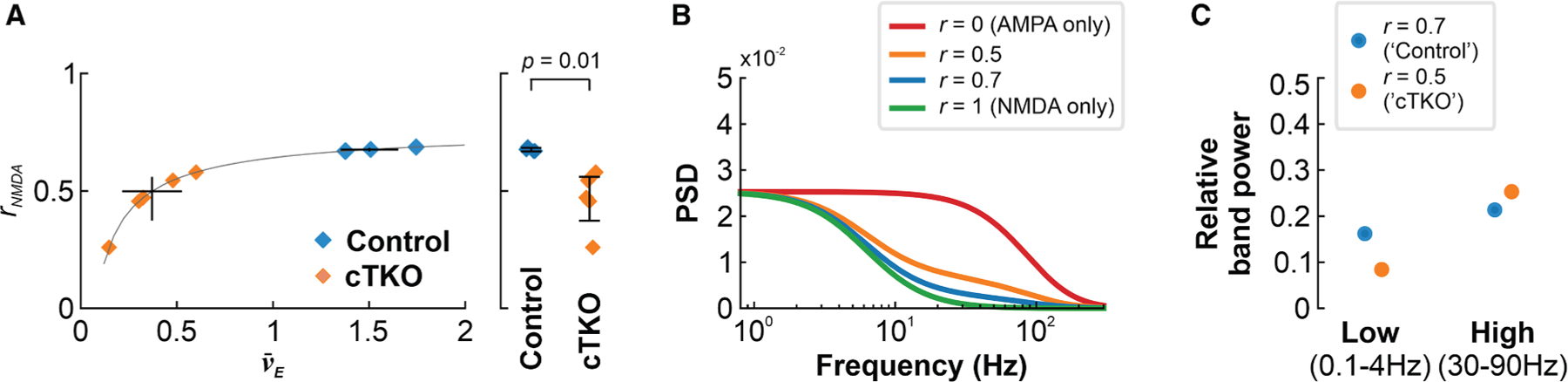
Computational modeling implicates NMDAR deficits in cTKO mice (A) (Left) Two-photon data (see Figures 3A–3C) from cortical layers 2/3 in control and cTKO mice were used to infer the fraction of NMDARs ($r_{NMDA}$, model-derived activity rate of excitatory population given by ${\bar{\nu }}_{E}$; see methods). (Right) cTKO mice were inferred to have a significantly smaller fraction of NMDARs, t test, t(7) = 3.5, *p* = 0.01. Each data point represents an individual animal (*N* = 4 controls, *N* = 5 cTKOs). (B and C) The theoretical excitatory kernel (see methods) was used to calculate the PSD for different ratios of NMDARs $\left(r_{NMDA}\right)$. Reduced $r_{NMDA}$, as inferred in cTKO mice, leads to a decrease in the relative band power at low frequencies and an increase at high frequencies, recapitulating that seen in empirical data from cTKO animals. See also Figure S4.

To investigate this, we first harnessed a mathematical model^47^ that is able to capture the activity profiles of both active and silent neurons (i.e., neurons that are undetectable to electrophysiological methods),^47,48^ within a balanced-state neuronal network composed of mixed synapses containing both AMPARs and NMDARs. Importantly, this analytically tractable model produces self-consistent solutions for diverse architectures, including both feedforward and recurrently connected populations, and provides predictions on the contribution of both receptor types to experimentally obtained neuronal activity profiles. Utilizing our two-photon Ca^2+^-imaging dataset during SWA (Figures 3A–3C), which comprised functionally silent and active neurons, Bayesian inference of model parameters revealed a potential invariability in one of these across both control and cTKO mice. This enabled us to analytically describe the dependence of NMDAR fractions on the mean neuronal activity profile. Notably, our computational modeling approach indicated that suppressed neuronal dynamics in cTKO mice relative to controls were indeed consistent with a reduced fraction of NMDARs (Figures 4A and S4A–S4C). Furthermore, we estimated the theoretical power spectral density (PSD) of the membrane potential using model-derived NMDAR fractions in control and cTKO mice (Figures 4B and 4C). These estimates were analogous to our experimental observations of a decrease in relative low-frequency band power, and increase in relative high-frequency band power, in cTKO mice versus controls, providing further theoretical support for the hypothesized NMDAR impairment in cTKO animals.

**Figure 5.**
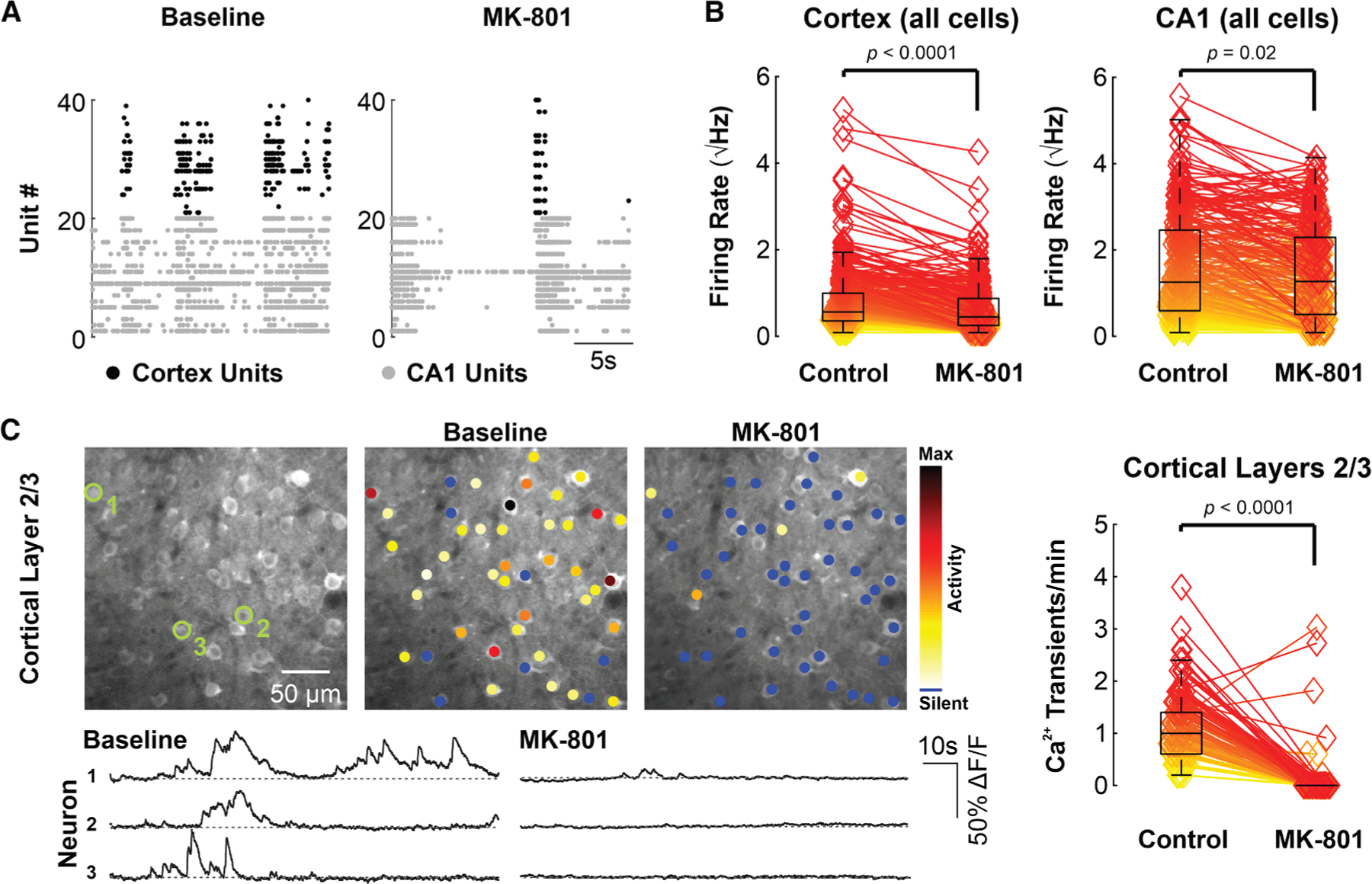
NMDARs support spontaneous neuronal activity *in vivo*, and NMDAR hypofunction induces functional phenotypes analogous to those observed in mice lacking the APP family (A) Example raster plots from *in vivo* Neuropixels recordings of 40 randomly selected cortical (black) and CA1 (gray) units during SWA in a control mouse during baseline (no drug, left) and 45 min after administration of the NMDAR antagonist MK-801 (1 mg/kg, i.p., right). (B) Comparison of neuronal firing rates in individual cortical and CA1 neurons (*N* = 328 and *N* = 269, respectively, from 4 control mice) before and 45 min after MK-801 administration, indicating a widespread hypoactive effect by NMDAR antagonism. Each data point pair and bridge represents an individual unit with warmer colors indicating higher baseline firing rates. Top and bottom of boxplots indicate 75th and 25th percentiles, respectively. (C) (Top left) Example *in vivo* two-photon fluorescence images of jRCaMP1b-expressing layer 2/3 cortical neurons in visual area of WT mice during baseline (no drug, top middle) and at 45 min after administration of the NMDAR antagonist MK-801 (1 mg/kg, i.p., top right). Colored markers indicate mean levels of spontaneous Ca^2+^ activity in individual neurons with cool colors indicating hypoactivity. (Bottom) Spontaneous Ca^2+^ activity (dF/F) from three example neurons circled and numbered in top left panel, before (left) and after (right) MK-801 administration. (Right) Quantification of effect of MK-801 on spontaneous Ca^2+^ activity in layer 2/3, indicating a pronounced suppression and increase in silent neuron fraction by NMDAR antagonism (*N* = 143 neurons from four animals). Each data point pair and bridge indicates an individual neuron with warmer colors indicating higher baseline Ca^2+^-transient rates. Top and bottom of boxplots indicate 75th and 25th percentiles, respectively. (B and C) *p* values obtained from LME models and provided as insets with statistical details provided in Table S5. See also Figure S4.

To experimentally test the notion that NMDARs contribute to spontaneous neuronal activity, we examined the effects of NMDAR antagonism in control mice through administration of the non-competitive NMDAR antagonist MK-801 (1 mg/kg, i.p., based on published standard protocols, see, e.g., Vesuna et al.^49^). Neuropixels recordings indicated that NMDAR blockade by MK-801 was indeed associated with a significant reduction in neuronal firing across all identified cells/units in visual cortex and CA1 45 min after administration (Figures 5A and 5B; Table S5). We additionally performed two-photon Ca^2+^-imaging using the red-shifted calcium indicator jRCaMP1b in WT mice during SWA, which enabled monitoring of neuronal activity in not only superficial layers 2/3, but also, in a subset of animals, in the comparatively less well explored layer 5. Here again, we similarly observed a drastic reduction in cortical neuron activity after 45 min, as well as numerous silent neurons across these layers (Figures 5C and S4D; Table S5). These experiments suggest that spontaneous neuronal activity in unaffected animals *in vivo* is, in part, dependent on intact NMDAR function, and that NMDAR antagonism induces a phenocopy of the functional abnormalities observed in cTKO mice, consistent with our theoretical modeling (see also Ruggiero et al.^50^).

### NMDAR deficits are salient in the absence of the APP family

**Figure 6.**
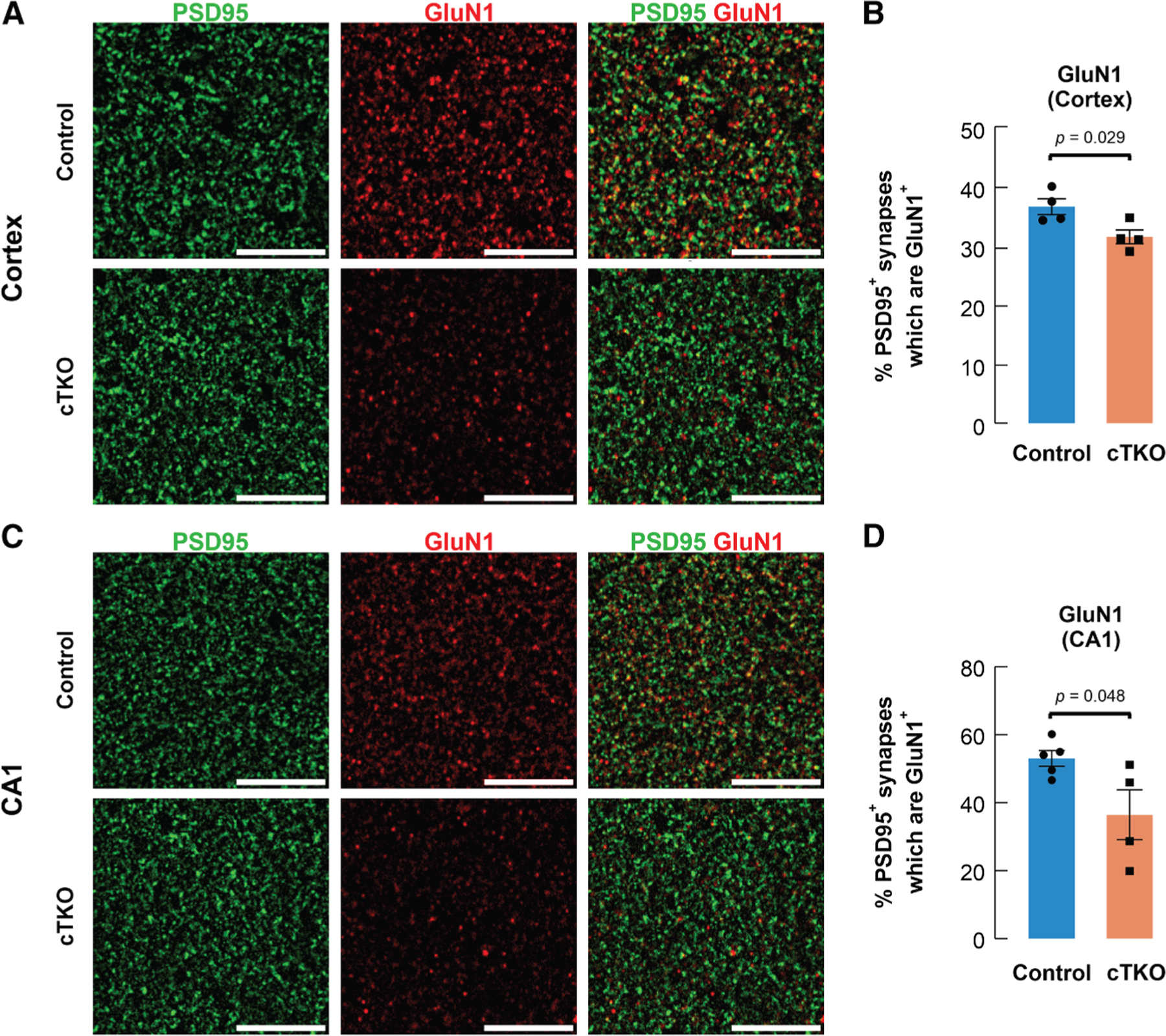
Reduced synaptic GluN1 NMDAR subunit in cortex and CA1 of cTKO mice (A) Immunofluorescence images showing PSD95 (green) and GluN1 (red) in cortex of control and cTKO mice. (B) Percentage of PSD95^+^ synapses that contain GluN1, indicating reduced synaptic GluN1 in cortex (retrosplenial and somatomotor) of cTKO mice compared with controls (each data point represents an individual animal, *N* = 4 control and *N* = 4 cTKO, error bars are SEM, t test, t(6) = 2.9, *p* = 0.029. (C) Immunofluorescence images showing PSD95 (green) and GluN1 (red) in medial CA1 of control and cTKO mice. (D) Percentage of PSD95^+^ synapses that contain GluN1, indicating reduced synaptic GluN1 in CA1 of cTKO mice versus controls (each data point represents an individual animal, *N* = 5 control, 4 cTKO, error bars are SEM, t test, t(7) = 2.4, *p* = 0.048. Scale bars, 10 μm. See also Figure S5.

Next, to establish whether NMDARs were altered in cTKO mice versus controls, we conducted *ex vivo* confocal imaging of synapses in cortical and CA1 slices. We first determined if there was improper localization of NMDA or AMPA receptor subunits to synaptic sites through fluorescent immunohistochemistry of different receptor subunits along with the postsynaptic marker PSD95. In the cortex (retrosplenial and somatomotor areas comparable with our two-photon dataset) of cTKO mice, there was a significant reduction in the percentage of PSD95^+^ puncta colocalized with the NMDAR subunit GluN1, which is essential for forming functional NMDARs^51^ (Figures 6A and 6B). In contrast, synaptic localization of AMPA receptor subunits GluA1 and GluA2 in cTKO mice was comparable with controls (Figure S5A). Within medial CA1 of cTKO mice, synaptic localization of GluN1 was also reduced, while localization of GluA1 and GluA2 was again similar to controls (Figures 6C, 6D, and S5B). We also examined total dendritic lengths and spine density in the cortex and found normal dendritic arborization albeit with a small but statistically significant decrease in spine density in apical and basal dendritic segments of layer 2/3 pyramidal neurons (Figures S5D and S5E). These data extend our previous report of slightly reduced spine density in basal and mid-apical dendritic segments of CA1 pyramidal neurons.^12^ Together, these findings suggest a reduction in synaptic NMDARs in cTKO mice that may impair their normal function.

### Restoring NMDAR function ameliorates functional and behavioral impairments associated with the loss of the APP family

**Figure 7.**
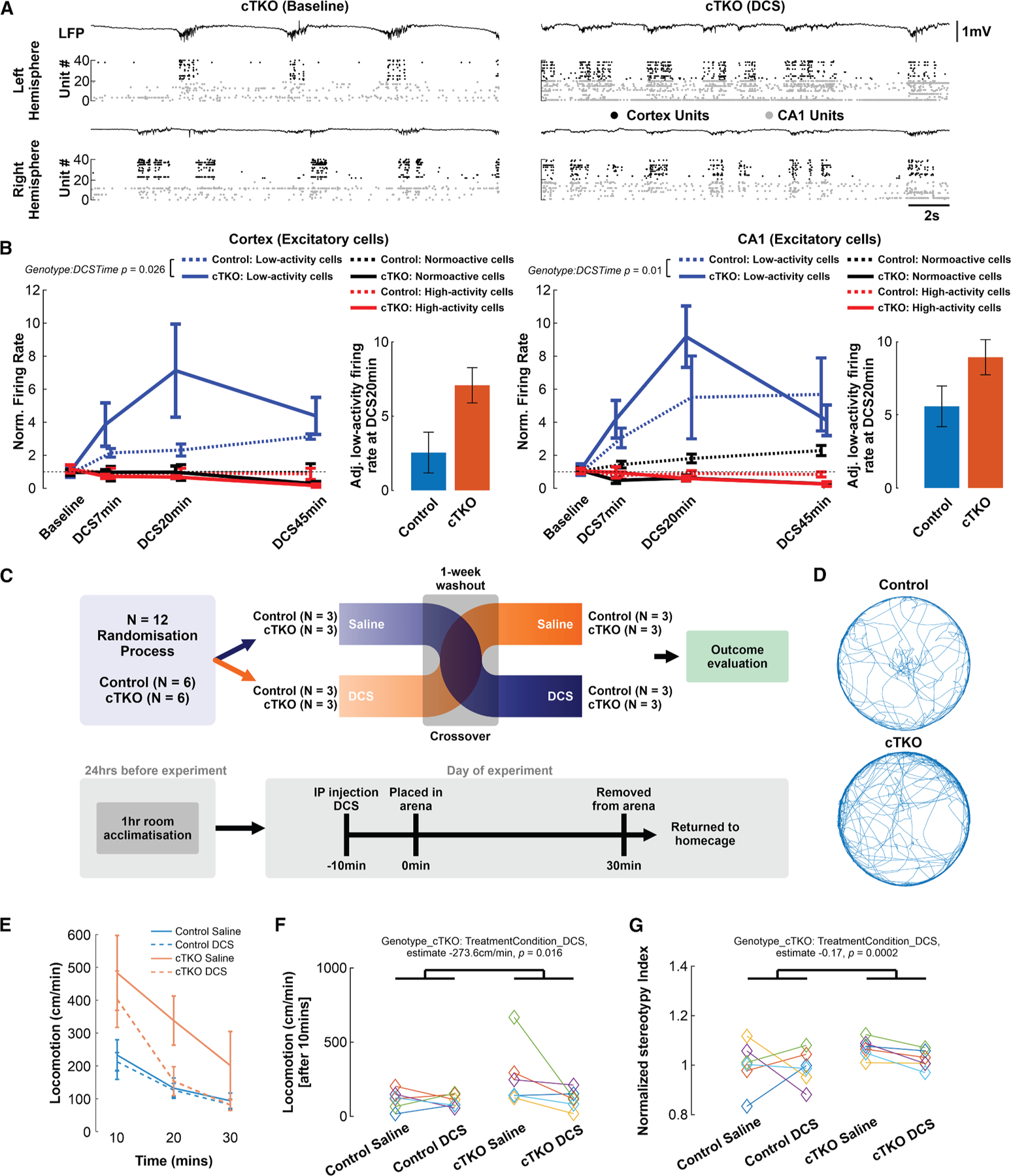
NMDAR agonism ameliorates functional neuronal impairments and behavioral deficits associated with loss of the APP family (A) Example raster plots from Neuropixels recordings of 40 randomly selected cortical (black) and CA1 (gray) units, and cortical LFP traces, in both hemispheres during SWA in a cTKO mouse under baseline conditions (no drug, left) and 20 min after the administration of the NMDA partial agonist DCS (30 mg/kg, i.p., right). (B) DCS treatment induced a significant and selective enhancement of firing rates in excitatory low-activity neurons of cTKO mice versus controls, in both cortex and CA1, with a distinctive non-linear treatment response that peaked at 20 min after DCS administration. Treatment response data over time represent mean normalized firing rates at the animal level (*N* = 3 control, *N* = 4 cTKO) with error bars as SEM. Inset bar graphs display LME model coefficient estimates, accounting for both fixed and random effects, with 95% confidence intervals (CIs) at 20 min after DCS (note no CI overlap between genotypes). Interaction *p* values obtained from LME models and provided as insets with full statistical details provided in Table S6. (C) Schematic describing open-field (OF) behavioral test protocol which incorporated a randomized, blinded, cross-over trial design to assess the impact of DCS on behavioral measures in control and cTKO mice (*N* = 6 per genotype). (D) Example movement paths of a control (top) and cTKO (bottom) mouse over 30 min within the circular OF arena. (E) Mean locomotor activity of cTKO mice in the OF arena over time was greater than control mice and normalized by DCS following a delay of approximately 10 min (i.e., 20 min following DCS administration). Error bars are SEM. (F and G) Quantification showing that DCS treatment specifically suppressed both locomotor hyperactivity and excessive stereotypy in cTKO mice relative to control animals and compared with the saline condition (each data point represents an individual animal, *N* = 6 control and *N* = 6 cTKO mice). Interaction *p* values obtained from LME models and provided as insets with full statistical details provided in Table S7. See also Figure S6.

To test the hypothesis that NMDAR hypofunction underpinned the observed functional and behavioral impairments in cTKO mice, rendering these more responsive to pharmacological targeting versus unaffected controls, we administered the NMDAR partial agonist D-cycloserine (DCS, 30 mg/kg, i.p., based on pilot experiments and published standard protocols, see, e.g., Won et al.^52^) during bilateral Neuropixels recordings of SWA. We recorded and tracked excitatory visual cortex and CA1 neuron firing rates at baseline (pre-DCS treatment, T = 0) and at 7, 20, and 45 min after DCS administration, and classified neurons according to their baseline firing rates (Activity Class: low activity <0.1 Hz and high activity >4 Hz, corresponding with the lower and upper bounds of slow-delta frequency bands, respectively). We then leveraged an LME model to investigate whether DCS treatment preferentially influenced neuronal firing rates in cTKO mice and to assess how effects varied across neuronal activity classes and DCS treatment time, considering both linear and non-linear trends, while accounting for inter-subject variability. This revealed a significant and selective DCS-induced increase, and non-linear response, in low-activity excitatory neuron firing in cTKO mice compared with controls, in both visual cortex and CA1, and peaking at approximately 20 min after DCS administration (Figures 7A and 7B; Table S6). Unexpectedly, we also found reduced cross-hemispheric coherence of cortical multi-unit activity in cTKO animals to be enhanced after DCS, an effect not observed in control mice (Figure S6A), suggesting that at least part of the fundamental deficit was associated with reduced neuronal dynamics in cTKO mice, and arguing against developmental structural abnormalities as the sole cause. The augmented effect of DCS in cTKO mice relative to controls is consistent with the notion that NMDAR function is conserved in unaffected control mice and already contributes strongly to ongoing activity. Notably, as an independent control experiment, administration of the AMPA receptor positive allosteric modulator CX546 at 20 mg/kg, i.p., (based on published standard protocols, see, e.g., Huang et al.^53^) in cTKO mice failed to elicit robust changes in firing rates (Figure S6B), suggesting an NMDAR-sensitive response decoupled from unspecific increases in neuronal excitability.

Lastly, we examined whether NMDAR agonism could also bring about behavioral improvements in cTKO mice. Since we previously observed locomotor hyperactivity and increased stereotypy (repetitive behavior) in cTKO mice during an open-field (OF) test,^12^ and such phenotypes have been linked to diminished NMDAR function,^54^ we adopted a randomized, blinded, cross-over trial design and assessed behavioral outcomes to the OF test after DCS or saline administration as a vehicle control (Figures 7C and 7D). NMDAR partial agonism by DCS reduced locomotor hyperactivity in cTKO mice to control levels (Figure 7E), with effects most prominently beginning 20 min after administration (i.e., after 10 min of being placed in the arena, and consistent with our functional data) (Figure 7B). LME modeling incorporating a fixed effect of treatment sequence (saline or DCS condition first) to account for potential carryover or order effects, and again controlling for inter-subject variability, demonstrated a significant interaction between genotype and treatment condition (Table S7). Importantly, this analysis indicated that DCS treatment attenuated both locomotor hyperactivity and excessive stereotypy in cTKO mice versus control animals, and relative to the saline condition (Figures 7F and 7G; Table S7). In addition, while a significant monotonic relationship between locomotor activity and stereotypy was also observed, this did not appear to be strongly modulated by genotype or treatment type (Figure S6C). Taken together, these results provide evidence for a functional and behavioral improvement in cTKO mice after NMDAR agonism, and, in so doing, further implicate NMDAR dysfunction in the absence of APP family members.

## DISCUSSION

Spontaneous fluctuations in neural activity, including that in single-neuron firing dynamics and population-level oscillatory rhythms, are an integral property of intact neural circuits *in vivo* and central to information processing and brain-behavior relationships.^21,55,56^ Here, we report that the absence of the APP family in excitatory neurons promotes a robust, yet rectifiable, attenuation of spontaneous local and distributed neural dynamics in both hippocampal and neocortical regions, as well as impairments of behavior. We observed strongly attenuated spiking dynamics, with increased low-activity/silent neurons across cortico-hippocampal circuits, and an impairment of low-activity neurons to respond flexibly to changes in behavior (i.e., locomotion), a key feature of this subpopulation of neurons that may support encoding and integration of novel information during behavior, and facilitate learning and memory consolidation.^35,36^ These changes at the single-neuron level occurred alongside an impairment of large-scale oscillatory activity, including reduced low-frequency activity and long-range coherence, as well as an increase of high-frequency oscillations. These findings provide insights that help to contextualize previous reports of multi-domain cognitive deficits in the absence of the APP family, and in which single-cell *in vitro* electrophysiology and structural deficits were subtle and restricted to hippocampus.^12,13^ In so doing, our findings implicate APP proteins in excitatory neurons as playing an important physiological role in shaping spontaneous neuronal activity, which represents a fundamental property of the intact brain *in vivo*.

Our observation, supported by multiple independent lines of evidence (computational modeling, confocal imaging, and pharmacological manipulation), that APP family members collectively influence ongoing neuronal activity via an effect on post-synaptic NMDARs, provides crucial *in vivo* context for a previously reported physical and functional coupling between the two elements *in vitro*. Consistent with our data, in transfected HEK cells and neurons, all three APP family members were found to biochemically interact with GluN1 and impact trafficking and cell surface expression of NMDARs, with reductions in APP decreasing NMDAR current density and surface levels^57–59^ (and see also Cousins et al.^60^). In turn, NMDAR activation itself decreases APP surface expression and modulates APP processing.^59,61,62^ While NMDARs are important for slow synaptic processes and plasticity, our results provide further evidence for a key role of NMDARs in supporting fast spontaneous neuronal activity *in vivo*,^50^ since antagonism of these in unaffected mice led to a marked impairment of spontaneous neuronal activity and increased neuronal silencing, which phenocopied changes observed in cTKO mice.

We note that a previous study using hippocampal slice recordings in a postnatal triple KO of the APP family demonstrated increased excitability in hippocampal neurons upon current injection, which was attributed to reduced expression of Kv7 channels that mediate potassium currents.^13^ Here, RNA-seq analysis revealed a reduction in Kv7 gene expression in the hippocampus, but not in the neocortex, where we conversely observed an impairment of neuronal function. At first sight, this reported increase in excitability may seem to contradict our findings of reduced neuronal dynamics. However, hippocampal neurons in slice preparations do not usually display spontaneous activity patterns such as those which manifest in the intact living brain across behaviorally relevant states. In addition, previous research has shown that blocking activity in neuronal cultures for 48 h increases neuronal sensitivity to subsequent current injections, partly due to changes in potassium currents.^63^ These results raise the possibility that the previously reported increase in neuronal excitability within slice recordings might be a consequence of suppressed neuronal circuit activity *in vivo*, similar to what we observed following APP family knock out.

Our findings, demonstrating a suppression of neuronal dynamics and hypoactivity in the absence of the APP family, appear to align inversely with reports that the overexpression of APP leads to neuronal hyperactivity in the same circuits. For example, network disruptions and increased spontaneous activity within cortical and hippocampal circuits have been reported in mouse models of AD overexpressing mutant human APP, as well as humans at early disease stages.^19,64,65^ While this has been attributed to the effects of increased downstream production of Aβ,^66,67^ other work, involving the overexpression of WT human APP and mutant human APP resistant to BACE cleavage, without an elevation in Aβ,^68,69^ have also reported a neuronal hyperactivity phenotype with APP gain of function. Notably, our findings provide experimental support for a previous hypothesis that APP overexpression, in itself, renders the brain more susceptible to hypersynchronous network activity by exacerbating the physiological function of the endogenous protein.^70^ It has also been recently shown that there is a potential cellular re-distribution of APP from somatic to extra-somatic sites in patients with, and mouse models of, AD,^71^ and future studies could examine whether there is also a redistribution of APP and its homologues away from postsynaptic sites, and whether this could impair the interaction with NMDARs (perhaps differentially by AD-related APP mutations), and contribute to suppressed activity such as that which we observed and which can be found in the AD brain with more advanced pathology.^19,72^ While the development of potential disease-modifying therapies in AD have focused on Aβ, and gained increased traction following the progress of recent clinical trials, it is noteworthy that there is ongoing discussion as to the importance of APP *per se* in the pathoprogression of the disorder.

In conclusion, the physiological role of the APP family has been sought since its first description more than 25 years ago; examination of KO animals for APP alone proved insufficient to infer its physiological role, since the homology of APLP1 and APLP2 proteins might substitute functionally.^73^ By exploiting a cTKO that occurs at an early developmental timepoint, we demonstrate a widespread alteration in neural system integrity at the cell, regional, and neural circuit levels with a marked impact on behavior. At least one effect of the loss of the APP family relates to synaptic localization, and hence function, of NMDARs on excitatory neurons, an effect that could be rapidly rectified pharmacologically. Our findings thus provide a richer picture of how the APP family impacts on neuronal and circuit function in the intact living brain,^65^ one that might also advocate a nuanced approach if this family is targeted for therapy in AD or related disorders, given potential effects on ongoing neuronal activity and behavior.

### Limitations of the study

In interpreting these findings, it is also important to consider limitations to the study. First, the APP family was deleted prenatally from approximately E11.5, which may introduce developmental effects that could differ from postnatal or adult-onset models; however, that pharmacological enhancement of NMDAR function ameliorated both circuit and behavioral phenotypes suggests that at least some deficits are functional and mechanistically reversible in the adult brain. Second, the KO was restricted to excitatory neurons, and potential contributions from inhibitory or non-neuronal cell types remain untested. Third, while our findings strongly implicate NMDAR hypofunction, other (untested) mechanisms downstream of APP family loss may also contribute to the observed functional and behavioral phenotypes which we observed.

## RESOURCE AVAILABILITY

### Lead contact

Further information and requests for resources and reagents should be directed to and will be fulfilled by the lead contact, Marc Aurel Busche (m.busche@ucl.ac.uk).

### Materials availability

This study did not generate new unique reagents.

### Data and code availability

- Source data for key quantitative comparisons presented herein will be deposited at Zenodo (https://doi.org/10.5281/zenodo.15210928) and made publicly available as of the date of publication.
- MATLAB code to reproduce key quantitative comparisons presented herein will be deposited at Zenodo (https://doi.org/10.5281/zenodo.15210936) and made publicly available as of the date of publication.
- Any additional information required to reanalyze the data reported in this paper will be available from the lead contact upon reasonable request.

## STAR★METHODS

### EXPERIMENTAL MODEL AND STUDY PARTICIPANT DETAILS

#### Mice

Mouse lines used in this study were generated in accordance with the guidelines and regulations set forth by the German Animal Welfare Act and the Regierungspräsidium Karlsruhe, Germany. Animals were housed in a temperature- and humidity-controlled, specific-pathogen-free facility in the same room with a 12 h/12 h light/dark-cycle in Makrolon Type II (360 cm^2^) cages with standard bedding, alone or in groups, and had *ad libitum* access to standard chow and water. Generation and genotyping of individual knockout mice were described previously: APP^flox^ and APLP2^flox84^; APLP1-KO^14^; NexCre.^28^ The mutant mouse lines with modifications of only one family member (APP^flox^, APLP2^flox^, APLP1-KO) had previously been backcrossed to C57BL/6 for more than 6 generations. The three strains were crossbred until the desired genotypes were generated. Final matings were APP^flox/flox^/APLP2^flox/flox^/APLP1^−/−^ x APP^flox/flox^/APLP2^flox/flox^/APLP1^−/−^ NexCre^+/T^ to obtain 50% NexCre cTKOs and 50% APLP1-KO littermates. APLP1-KO littermates were used as internal controls in this study since APLP1-KO mice have previously undergone behavioral testing and showed no impairments in tests of learning and memory^30^ nor do they display altered neuronal activation patterns compared to C57/BL6 wild-type mice (Figure S7). Importantly, NexCre cTKO mice showed very poor breeding performance with impaired mating behavior and disturbed maternal/pup interaction, consistent with impairments in sociability, and impaired neonatal vocalizations.^12^ To circumvent these breeding problems, animals in this study were generated by *in vitro* fertilization and embryo transfer into outbred foster mothers.^12^ Constitutive single knockout mice and wild-type counterparts used in this study have been described previously (APP-KO, APLP1-KO, APLP2-KO).^14,77^ Animals of both sexes were shipped to the Massachusetts General Hospital and McLaughlin Research Institute for Biomedical Sciences and University College London (UCL) for functional and behavioral experiments and housed under similar conditions after arrival. Imaging experiments shown in Figures 3A–3D, S3D, and S7 were approved by the Institutional Animal Care and Use Committees of the Massachusetts General Hospital and McLaughlin Research Institute for Biomedical Sciences. All surgical and experimental procedures for Figures 1, 2, 3E–3H, 5, 6, 7, S2, S3A–S3C, S3E, S4D, and S6 were conducted in accordance with the Animal (Scientific Procedures) Act 1986, approved by the Animal Welfare and Ethical Review Body (AWERB) at UCL, and performed under an approved UK Home Office project license at UCL. All mice were housed in a temperature- and humidity-controlled specific pathogen free facility and maintained in a 12-h light/dark cycle with food and water supplied *ad libitum,* alone or in groups, with standard cages and bedding until surgical procedures. All mice were healthy, immunocompetent and drug and procedure naive prior to surgical procedures. Following surgical procedures, mice were individually housed in cages to prevent interference of cranial windows and head-fixation apparatus by cage-mates and checked daily for clinical signs until recordings. Both male and female C57/BL6, APP-KO, APLP1-KO, APLP2-KO and cTKO mice aged between 7 and 15 months of age, with an additional cohort of wild-type mice aged 3–5 months, and a cohort of 10-month-old BTBR T^+^ Itpr3^tf^/J (BTBR) mice, were used for experimental procedures in this study. Mixed-sex cohorts were used throughout this study to reduce potential sex-related bias; however, we did not test for sex differences and therefore cannot exclude that sex may influence our results and which represents a potential limitation regarding generalizability. All animals were randomly allocated to the experimental groups. Sample sizes were determined according to standards in the field and previously published research.

### METHOD DETAILS

#### Surgical procedures for *in vivo* imaging

Mice for imaging experiments shown in Figures S3A–S3D and S7 were anesthetized with ∼4 % isoflurane in oxygen (O_2_) and maintained on ∼2 % isoflurane during surgical procedures. To maintain a consistent body temperature of approximately 37 °C, a heating pad was used, and ophthalmic ointment was applied to protect the eyes during procedures. Aseptic techniques were strictly followed as the skin above the skull was carefully excised, and two craniotomies were performed over the left and right occipito-parietal cortices (centered at approximately −2.7 mm AP ±2.7 mm ML), using a fine-tipped dental drill. Next, the genetically encoded fluorescent Ca^2+^ indicator GCaMP6f (pAAV.Syn.GCaMP6f.WPRE.SV40^76^; Addgene viral prep # 100837-AAV1) was injected into cortical layer 2/3 at a rate of 0.2 μL min^−1^ using a stereotactic device (Kopf Instruments) and a Pump 11 Elite microsyringe pump (Harvard Apparatus). Each cortical hemisphere received a single injection of ∼1 μL viral construct. A single round glass coverslip (8 mm diameter) was placed over both craniotomies and sealed to the skull using a mixture of dental cement (Jet) and cyanoacrylate glue. After surgery, mice were returned to their home cage and analgesia (buprenorphine and acetaminophen) was provided for three days postoperatively.

Mice for experiments in Figures 1, 5C, and S4D were anesthetized with isoflurane (3–4 % induction, 1.5–2 % for maintenance) and provided with subcutaneous carprofen for pain relief during subsequent surgery procedures. Eye ointment (Viscotears) was applied to protect the eyes during procedures and the animal’s head was shaved to remove fur. The animal was subsequently secured in a stereotaxic frame (WPI) and over a heating blanket to maintain normothermia at 37 °C during surgery. Diluted chlorhexidine was used to disinfect exposed skin and cleaned with ethanol, after which lidocaine cream was applied to the planned incision area using a sterile applicator. All surgical procedures were performed under aseptic conditions and anesthetic depth was monitored throughout via the pedal reflex and breathing rate. Subcutaneous saline (0.2 mL) was provided every hour of surgery to maintain hydration. The skin overlying the skull was carefully retracted following a small incision, and skull fascia gently removed using forceps. For subsequent awake two-photon (2P) recordings, a small craniotomy overlying retrosplenial cortex (RSC, centered at the midline −2 mm AP) was performed using a hand-held microdrill (OmniDrill35, WPI) and 1 μL of the genetically encoded fluorescent Ca^2+^ indicator jGCaMP8s (pGP-AAV-*syn*-jGCaMP8s-WPRE^74^; Addgene viral prep # 162374-AAV1) injected into layer 2/3. For 2P recordings under anesthesia, a small craniotomy overlying occipito-parietal cortex (centered at approximately −2.7 mm AP ±2.7 mm ML) was also performed using a hand-held microdrill (OmniDrill35, WPI), and 1 μL of the genetically encoded fluorescent Ca^2+^ indicator jRCaMP1b (pAAV. Syn.NES-jRCaMP1b.WPRE.SV40^75^; Addgene viral prep # 100851-AAV1) injected into cortical layers 2/3 or cortical layer 5. All craniotomies were performed under a surgical microscope (Leica) and constant cooling with sterile PBS, and all indicators were injected at a rate of 0.1 μL min^−1^ using a 10 μL Hamilton Syringe (Hamilton Company) secured to a microsyringe pump (UMP3 UltraMicroPump, WPI). A small glass coverslip was then placed over the craniotomy, and dental cement applied to secure the cranial window and to cover any remaining exposed skull. For subsequent 2P recordings under awake conditions, we additionally affixed an aluminum headplate (for head-fixation) with a 7 mm imaging well surrounding the craniotomy with dental cement (Super-Bond C&B, Sun Medical). After surgery, mice were provided with subcutaneous buprenorphine for immediate pain relief and returned to their home cage, with carprofen provided in drinking water overnight and continued for three days postoperatively. Mice were allowed to recover for at least two weeks before imaging.

#### *In vivo* calcium imaging and analysis

For all imaging experiments under anesthesia, mice were initially anesthetized with isoflurane (3–5 % induction) and placed in a stereotaxic frame and over a heating blanket to maintain body temperature at ∼37 °C throughout experimental procedures. Subsequent recordings were made under light (∼1 %) isoflurane anesthesia. For all imaging experiments in awake animals, mice were extensively habituated to handling and head fixation over several days and progressively increasing time periods until comfortable within their environment. Mice were subsequently head-fixed for imaging sessions, with their body supported by a custom-made 3D printed tube. Behavior was monitored throughout imaging using an infrared camera (Point Gray Chameleon3).

Imaging experiments shown in Figures 3A–3D and S7 were performed using a Fluoview FV1000MPE multiphoton microscope (Olympus) controlled by Fluoview software (Olympus), and equipped with a mode-locked MaiTai Ti:sapphire laser (Spectra-Physics) and a 25X, 1.05 NA, Olympus water immersion objective. We selected the hemisphere (out of the two injected with the Ca^2+^ indicator) exhibiting superior viral expression for subsequent 2P imaging. To image spontaneous fluorescence signals from GCaMP6f-expressing cortical layer 2/3 neurons, a wavelength of 900 nm was used, and images recorded at ∼15 Hz with a resolution of 256 × 256 pixels. The recorded images were analyzed offline using the Fiji package of ImageJ^80^ (National Institutes of Health) and Igor Pro (WaveMetrics). First, regions of interest (ROIs) were drawn around individual neuronal somata; then, relative GCaMP6f fluorescence change versus time traces were generated for each region of interest. Ca^2+^ transients were identified by changes in relative fluorescence that were three times larger than the standard deviation of the baseline fluorescence. Transients were counted and divided by the total recording time in minutes for each experiment to yield calcium transient rate per minute.

Experiments presented in Figures 1, 5C and S4D were performed using a custom-built resonant-scanning two-photon microscope (Independent NeuroScience Services) controlled by ScanImage (Vidrio) and equipped with a femto-second pulsed laser (Coherent Chameleon Discovery NX) and a 16X, 0.8 NA, Nikon water immersion objective. Power on sample did not exceed 50 mW. Images were acquired at 30 Hz frame rate with a resolution of 512 × 512 pixels. Image data analysis was performed with Suite2p^78^ and custom MATLAB (The MathWorks) and Python (Python Software Foundation) scripts. The recorded image stacks were loaded into Suite2p for motion correction, cell soma ROI detection, and Ca^2+^ signal extraction. For imaging data acquired under awake conditions (with jGCaMP8s, Figure 1), we used the “anatomical only” segmentation method in Suite2p, which applies the Cellpose algorithm,^79^ to detect neurons purely based on their anatomy, rather than their activity, in order to avoid biasing our estimation of firing rates toward active neurons. Segmentation results were manually vetted to remove any non-somatic ROIs. ΔF/F traces were computed by normalizing each frame of each neuron by a 30 s rolling mean of the neuron’s activity. Firing rates at each frame were estimated from ΔF/F traces by applying the CASCADE deconvolution algorithm as previously described.^32,33^ Overall firing rates reported in Figure 1 were computed by summing each frame’s firing rate and dividing by the length of the recording(s). Ca^2+-^transient rates in Figure S2A were defined as the number of transients in the deconvolved trace per second with a peak above 0.2. Animal movement (Figure S2B) was calculated by binarizing the infrared video recording to silence the background and highlight the body of the mouse. We then cropped the image around the body of the mouse and computed the absolute value of the first differential of each pixel for each frame - yielding a value of 1 when movement was detected in the pixel. We then summed the differential across all pixels and applied min-max normalization to scale the value between 0 and 1. Movement epochs were defined as periods in which this value exceeded 0.1. Imaging of jRCaMP1b-expressing neurons in cortical layers 2/3 and 5 (Figures 5C and S4D) under light anesthesia was performed before and 45 min following intraperitoneal administration of the NMDA antagonist MK-801 (1 mg/kg). For each detected ROI (putative cell somata), the neuropil corrected signal was extracted by subtracting the neuropil fluorescence signal surrounding the ROI (F_n_) from the raw fluorescence signal within the ROI (F): F_corr_(t) = F(t)-0.7*F_n_(t). The baseline fluorescence (F_0_) was estimated by using robust mean estimation and relative fluorescence changes (ΔF/F=(F_corr_(t)-F_0_)/F_0_) over time generated for each ROI. Ca^2+^ transients were identified as relative changes in ΔF/F that were two times larger than the standard deviation of the noise band. Transients were again counted and divided by the total recording time in minutes for each experiment to yield calcium transient rate per minute.

Widefield imaging experiments shown in Figure S3D were performed using a high-speed EMCCD camera (Andor Luca) coupled to a BX61WI Olympus microscope equipped with a 2X, 0.14 NA, Olympus air objective. The GCaMP6f-expressing cortical surface was excited using a 120 W mercury arc lamp (X-Cite, Excelitas), and fluorescence images (251 × 250 pixels, 4 × 4 binning) acquired at ∼20 Hz using Micro-Manager.^85^ Raw fluorescence images were extracted for each recording trial (500 frames, trials exhibiting signal dropouts/discontinuities were disregarded) and a rectangular mask symmetrical about the midline applied. Three regions of interest (ROIs, 20 × 20 pixels) centered at the midpoint of each hemisphere were then identified at the top, middle, and bottom of the FOV. Timeseries for all pixels in each ROI were subsequently averaged, detrended and converted to ΔF/F (baseline fluorescence estimated as the 15^th^ percentile of the signal) and smoothed using a 2-point moving average filter. Within hemisphere synchrony was quantified by subjecting signals from anterior and posterior ROIs in each hemisphere to a mean phase coherence algorithm (MPC, see below), while cross-hemispheric synchrony was assessed by subjecting signals from central ROIs in both hemispheres to the same.

#### Surgical procedures for Neuropixels recordings

All surgical and experimental procedures were conducted in accordance with the Animals (Scientific procedures) Act 1986, approved by the Animal Welfare and Ethical Review Body (AWERB) at UCL, and performed under an approved UK Home Office project license at UCL. Mice were maintained in a 12-h light/dark cycle with food and water supplied *ad libitum*. Before beginning surgical procedures, mice were anesthetized with isoflurane (3–4 % induction, 1.5–2 % for maintenance) and provided with subcutaneous carprofen for pain relief during subsequent surgery procedures. Eye ointment (Viscotears) was applied to protect the eyes during procedures and the animal’s head was shaved to remove fur. The animal was subsequently secured in a stereotaxic frame (WPI) and over a heating blanket to maintain normothermia at 37 °C during surgery. Diluted chlorhexidine was used to disinfect exposed skin and cleaned with ethanol, after which lidocaine/prilocaine cream was applied to the planned incision area using a sterile applicator. All surgical procedures were performed under aseptic conditions and anesthetic depth was monitored throughout via the pedal reflex and breathing rate. Subcutaneous saline (0.2 mL) was provided every hour of surgery to maintain hydration. The skin overlying the skull was carefully retracted following a small incision, and skull fascia gently removed using forceps.

For acute Neuropixels recordings under anesthesia, two small craniotomies overlying bilateral visual cortices were performed using a hand-held microdrill (OmniDrill35, WPI) under a surgical microscope (GT Vision) and constant cooling with sterile PBS. A chlorided silver wire (acting as a ground/reference electrode) was gently inserted into a small skull indentation made over the cerebellum and secured using cyanoacrylate glue, and a cranial well created to encircle both craniotomies using dental cement (Jet). On completion of surgical procedures, these animals were transferred directly to the recording rig for experiments with no interruption of isoflurane anesthesia.

For awake Neuropixels recordings, the exposed skull was covered with a thin layer of veterinary glue (Vetbond). After drying, a layer of ultraviolet (UV) curing glue (Norland Optical Adhesive 81) was gently applied using a plastic pipette tip and briefly cured using a handheld LED UV curing spot lamp (Intertronics). A small craniotomy overlying visual cortex was subsequently performed using a hand-held microdrill (OmniDrill35, WPI) under a surgical microscope (Leica) and constant cooling with sterile PBS. A custom-built titanium head-fixation plate was then placed behind lambda and held in place using a custom-built mounting arm (Thorlabs). High-strength dental cement (SuperBond, Prestige Dental) was then applied to secure the plate to the skull overlying the cerebellum and create a well to encircle the craniotomy and cover any remaining exposed skull. On completion of surgical procedures, craniotomies were covered with a layer of sterile PBS and the entire well sealed with silicone elastomer sealant (KwikCast). Subcutaneous injection of buprenorphine (0.1 mg/kg) was subsequently administered for immediate post-surgical pain relief, and the animal allowed to recover from surgical anesthesia on a heated plate (37 °C) and monitored continuously. On recovery, animals were returned to the holding room in single-housed conditions and carprofen provided in drinking water overnight and continued for three days, during which the animal’s health and welfare was closely monitored.

#### Neuropixels recordings

Awake Neuropixels recordings (Figure 2) were performed at least one week following recovery surgery procedures and after habituation of the animal to handling, head-fixation apparatus and the experimental rig over at least three daily sessions and until the animal appeared undisturbed and comfortable with their environment. On experimental days, mice were carefully head-fixed to a holder which was elevated above a rolling Styrofoam wheel. After inspection of the structural integrity of the head-plate and cranial well, a retractable nose cone was gently positioned over the snout and the animal lightly sedated with isoflurane (∼1%) for subsequent cleaning of the cranial well, and implantation of Neuropixels probes^86^ (IMEC). After cleaning of the well surface using alcohol (70 % ethanol), the silicon cap was carefully removed with fine forceps and the underlying craniotomy covered with sterile PBS. A silver chloride ground/reference electrode was affixed to the edge of the cranial well and head-plate using dental cement (Jet) such that it contacted the PBS reservoir and an area of skull distal to the craniotomy.

For all experiments, Neuropixels probes were attached to a micromanipulator (QUAD, Sutter Instruments) and spatially referenced to visible bregma, and stereotactically maneuvered to ∼ −3 mm anterior-posterior (AP) and +/−3.26 mm medial-lateral (ML), orthogonal to the anterior-posterior (AP) axis and at 60 ° elevation, and overlying primary visual cortices. Neuropixels probes were unilaterally or bilaterally implanted at a rate ∼5–10 μm/s under remote micromanipulator control and visualized under a microscope (GT Vision). Following successful implantation, the brain was allowed to rest for at least 45 min before recordings, during which isoflurane anesthesia was either removed for awake experiments or, for acute non-recovery recordings, gradually lowered to maintain adequate anesthetic depth and promote physiological cortical activity levels. Following recordings, all animals were immediately sacrificed, and without recovery in acute anesthetic experiments, for subsequent lightsheet or immunohistochemical analysis.

Neuropixels recordings (∼30 kHz sampling rate) of spontaneous spiking activity were acquired using SpikeGLX software and processed and automatically spike sorted in Kilosort3^81^ using default settings. The brain regions traversed by each Neuropixels probe within each hemisphere, and location along the probe shank, were estimated using the experimental stereotactic coordinates (anterior-posterior, medial-lateral, micromanipulator azimuth and elevation) and custom-written MATLAB scripts incorporating a modification to the open-source Neuropixels trajectory explorer with the Allen Common Coordinate Framework (CCF) version 3 mouse atlas (https://github.com/petersaj/neuropixels_trajectory_explorer) (GPL-3.0 License). This yielded a predicted anatomical trajectory of the Neuropixels probe as a function of probe shank channel. As probes were not fully implanted (∼3200–3400 μm), an offset to this trajectory vector was computed by designing a depth template of channels, where cortical areas and CA1 regions were nominally set to a non-zero value and all other areas/channels set to zero. As a result, the depth template comprised of two square waves, reflecting the spread of cortical and CA1 regions according to the predicted trajectory, which was separated by the intervening corpus callosum. We next extracted unit incidence, unit firing rates and unit amplitudes, as well as total power in the ripple frequency band in the local field potential over time (110–250 Hz, elevated in CA1) (Figure S2C). Unit incidence and ripple power across channels/depths were normalized between 0 and 1 and smoothed using a moving average filter, with the product of both factors subsequently cross-correlated to the depth template, and the estimated offset between these given by the normalized cross-correlation possessing the largest absolute value (MATLAB function ‘finddelay’). This offset was then used to align the predicted anatomical trajectory to functional data, with small manual adjustments if necessary, and assign each neuron/unit to a putative brain-region, with this approach closely corresponding to anatomical validation by lightsheet imaging (described below and example in Figure S2C, left). We next restricted the putative bounds of interest in CA1 for further analysis by nominally selecting 150 μm either side of the hippocampal ripple power peak, rounded to the nearest inflection point in the unit incidence depth profile (examples in seven animals shown in Figure S2C, right).

#### Clustering of units into putative excitatory and inhibitory neuron classes

Spike waveforms for each identified neuron/unit were extracted, averaged across spiking events, and normalized. The first and second principal components were computed for each waveform (MATLAB function ‘pca’) and used as inputs to generate a Gaussian mixture distribution model (GMM, MATLAB function ‘fitgmdist’, 200 replicates) for subsequent clustering with two components (MATLAB function ‘cluster’), with the smallest cluster, consisting of artifactual signals, excluded from further analysis. Remaining waveforms were subsequently visually checked to confirm physiological appearance in PHY software (https://github.com/cortexlab/phy). Clustering of cells/units into putative excitatory and inhibitory neuron classes was performed in a standard manner using spike waveform properties (width of the spike deflection and trough-to-peak time)^87^ and fitting an unsupervised Gaussian mixture model (GMM) to the data using the iterative Expectation-Maximization (EM) algorithm (MATLAB function ‘fitgmdist’, 500 replicates, 500 iterations). We set the number of components/clusters to four based on previous reports^88^ and subsequently visually confirmed clustering efficacy. Putative fast-spiking inhibitory neurons were defined as those belonging to a distinct cluster associated with more brief ‘thin’ waveform dynamics (corresponding to physiologically plausible ∼10 % of total units in awake and anesthetized experiments) while remaining units were considered to be primarily comprised of excitatory neurons. Neurons/units were defined as well isolated and included for further analysis if they met the following criteria: amplitude cut-off <0.1 (“unit completeness”) and inter-spike-interval violations <0.5 (“unit contamination”).

#### Analysis of neuronal firing dynamics across states

Neuronal firing rates were calculated for each unit/neuron and collated. For awake experiments, we leveraged behavioral monitoring data (infrared camera and rotary encoder) to identify resting state and locomotion periods. Video data (30Hz sampling rate), capturing the head side-profile and forelimbs, was imported into Facemap^20,82^ to extract pupil size and face keypoints (pupil sizes subsequently normalized to the distance between nose-tip and anterior point of the eye in order to account for slight differences in camera positioning across animals). Rotary encoder data was converted to absolute rotations per minute (RPM) (thus including both forward and reverse locomotion), with timepoints during which angular acceleration was <0.025 RPM/sec and velocity <0.01 RPM defined as stationary periods, so as to disregard slow drift in the instrument. Locomotion data was then converted to speed using the diameter (15 cm) of the Styrofoam wheel. To further identify paroxysmal animal movements not related to locomotion (e.g., whisking, nose twitching, saccades, grooming, etc.), we calculated the rectified first approximate derivative of the mean intensity across all pixels for each frame in the video (resampled to match rotary encoder data sampled at 20 Hz), and defined signal crossings above 6 x median absolute deviations to be non-locomotor movement. We then identified 2 s time-windows across the entire recording period that were uninterrupted by locomotion or non-locomotor movement and defined these as resting state epochs. Active locomotion epochs were identified and defined as periods when locomotion remained above the stationary threshold for a minimum of 2 s. Resting-state and locomotion epochs for each animal were then used for calculation of neuronal firing dynamics for each state condition across consecutive experimental sessions (2–6 sessions of length 5–10 min) (Figure 2). We further calculated the normalized mean firing rate (MFR) modulation^26^ of individual neurons due to locomotion (modulation index, MI) by calculating (MFR_*locomotion*_ - MFR_*rest*_/MFR_*locomotion*_ + MFR_*rest*_). For prolonged pharmacological experiments under light anesthesia presented in Figures 5A, 5B and 7B, we concatenated consecutive experimental sessions (taken at baseline and 7-, 20- and 45-min following drug administration, each of 10 min duration) so as to track single neuron activity during treatment. Neuronal data at baseline (i.e., initial 10 min recording prior to drug treatment) for comparison between littermate controls and cTKO animals are presented in Figures 3E–3H and S3A. Supplementary data for other genotypes presented in Figures S3B and S3E were acquired during a 10min recording session in the absence of any intervention. Where it was not possible to concatenate experimental sessions during treatment due to artifact (Figure S6B), data is presented as animal averages at each treatment timepoint.

#### Analysis of local field potential and multi-unit activity

Local field potential (LFP) data was acquired at ∼2.5 kHz. We obtained frequency power estimates of within-hemisphere spontaneous LFP activity in awake (across resting epochs) and anesthetized states (across all recording time) using the multi-taper approach (‘mtspectrumc’, Chronux toolbox^83^). For cortex, we computed the median of the obtained power spectrum across cortical channels within each hemisphere. For hippocampus CA1, the power spectrum was taken from the channel which displayed the largest ripple band (110–250 Hz) power within the CA1 bounds, and presumed to colocalize with the pyramidal layer. This yielded a unitary estimate for each animal and hemisphere (averaged across hemispheres for bilateral recordings). Relative power of specific frequency bands was calculated by integrating the associated area using the Simpson’s rule (parabolic approximation) and as a fraction of that of the entire signal (<250Hz), with exclusion of the mains noise band 49–51 Hz. Cross-hemispheric cortical synchrony in bilateral recordings during slow wave activity was examined using a custom-written mean phase coherence (MPC) algorithm (described in detail previously, see^89,90^) that is not dependent on the amplitude of the input signals and is insensitive to constant phase delays between two signals such as those arising due to neural transmission latency between two hemispheres. Coherence analysis (average over 90 s windows with 30 s overlap) was conducted for each animal using non-spike-sorted multi-unit activity traces from each hemisphere (MUA, all neuronal spiking events across cortical channels aggregated and binned in 10 ms intervals, and normalized between 0 and 1). Cortical MUA traces during slow wave activity were also used to examine UP state differences between genotypes, following previous analytical protocols.^91^ Briefly, MUA activity traces (10 ms bins) for each animal were smoothed using a 100 ms Gaussian window prior to min-max normalization, and the distribution of MUA fitted using a Gaussian mixture model employing an iterative Expectation-Maximization algorithm (MATLAB function ‘fitgmdist’ with two Gaussians), with means and variances, ${\text{μ}}_{UP\text{-}MUA}$ and ${\text{μ}}_{DOWN\text{-}MUA}$, and ${\text{σ}}_{UP\text{-}MUA}$ and ${\text{σ}}_{DOWN\text{-}MUA}$, representing the cortical UP and DOWN state, respectively. The threshold for detection of an UP or DOWN state was defined as ${\text{μ}}_{UP\text{-}MUA}–{\text{σ}}_{UP\text{-}MUA}$ and ${\text{μ}}_{DOWN\text{-}MUA}+{\text{σ}}_{DOWN\text{-}MUA}$, respectively. The fraction of total time that MUA exceeded the UP state threshold during the course of the recording, and the average dwell time in transitory UP states, was subsequently calculated for each animal.

#### Drug intervention protocols

Stock solutions of MK-801 (Sigma), D-cycloserine (DCS, Cambridge Bioscience), and CX546 (Tocris) were produced, aliquoted and stored for up to 1 month at −20 °C. On the day of experiment, vials were allowed to thaw at room temperature prior to administration via intraperitoneal injection using a 26-gauge needle; MK-801 at 1 mg/kg,^49^ DCS at 30 mg/kg^52^ and CX546 at 20 mg/kg.^53^

#### Brain clearing and light-sheet fluorescence microscopy

Brain tissue samples were fixed in 4 % paraformaldehyde (PFA) overnight on a shaker at 4 °C and subsequently cleared using a modified immunolabeling-enabled three-dimensional imaging of solvent-cleared organs (iDISCO) clearing method.^92^ The fixed samples were washed three times in PBS for 30 min each, then dehydrated progressively in incrementally increasing concentrations of methanol in H_2_O (20 %, 40 %, 60 %, 80 %, 100 %) for 1 h each. The sample was then incubated in 66 % dichloromethane (DCM, Sigma)/33 % methanol solution at room temperature overnight on a shaker. The samples were subsequently washed twice in 100 % methanol for 1 h each, then bleached with chilled fresh 5 % H_2_O_2_ in methanol (1:5 dilution of 30 % H_2_O_2_ with methanol) overnight at 4 °C. Samples were then incubated in a 66 % DCM/33 % methanol solution at room temperature for 3 h on a shaker, and further incubated in 100 % DCM for 15 min twice on a shaker to wash out the methanol. Samples were transferred to 5 mL tubes (Eppendorf) filled with dibenzylether (DBE, Sigma) to prevent oxidation of the samples by the air and stored in DBE until imaging experiments.

Cleared brain tissue from a subset of animals, in which probes were coated with DiI (Vybrant CM-DiI Cell-Labelling Solution, ThermoFisher) prior to implantation, were imaged using a custom-built mesoscale selective plane illumination microscope (meso-SPIM).^93^ Tissue was immersed in DBE solution within a small quartz cuvette and secured using a 3D-printed holder, and fluorescence images acquired using Olympus MVX-10 macroscope at 1X magnification (voxel size 6.55 × 6.55 × 5 μm). Autofluorescence was captured using 488 nm laser excitation (LBX-488-200-CSB-OE 200 mW diode laser, Oxxius) and a 520/35 nm bandpass emission filter, and DiI fluorescence (representing probe tracks) captured using 561 nm laser excitation (LCX-561-200-CSB-OE: 561 nm 200 mW DPSS laser, Oxxius).

#### Immunohistochemistry

Brains were extracted from animals after intracardiac perfusion of phosphate-buffered saline (PBS; Gibco), embedded in optimal cutting temperature compound (OCT; CellPath) and frozen in liquid nitrogen. Sagittal sections of 10 μm thickness were cut using a Leica cryostat. Sections were fixed in 4 % paraformaldehyde (PFA) in PBS for 30 min prior to immunolabelling. Sections were blocked in blocking solution (10 % goat serum, 0.3 % Triton in PBS) for 3 h, incubated with primary antibodies in blocking solution overnight at 4 °C then secondary antibodies for 2 h at room temperature. Sections were then labeled with DAPI and mounted with ProLong Gold (ThermoFisher). Primary antibodies: PSD95 (1:1,000; Abcam; ab2723), GluN1 (1:1,000; Synaptic Systems; 114 103), GluA1 (1:500; Abcam; ab31232), GluA2 (1:1,000; Synaptic Systems; 182 105). Secondary antibodies: Goat anti-mouse 488 (1:1,000; Invitrogen), Goat anti-mouse 647 (1:500; Invitrogen), Goat anti-rabbit 594 (1:1,000; Invitrogen), Goat anti-guinea pig 488 (1:1,000; Invitrogen).

#### Imaging of immunohistochemical samples and analysis

Immunolabelled sections were imaged on a Zeiss LSM880 confocal microscope using 63X magnification (field of view (FOV): 134.95 μm by 134.95 μm, 1024 × 1024 pixels). Cortex: 1–2 regions (consisting of 3–7 FOV comprising entire thickness of cortex) at bregma = 0 and bregma = 2 from each of 2–3 sections per animal were imaged (minimum 4 regions per animal; results were averaged to yield a single value per animal). CA1: 1–3 FOV from each of 2–3 sections per animal were imaged (minimum 4 FOV per animal; results were averaged to yield a single value per animal). Object-based colocalization analysis was automated using the FIJI build of ImageJ software (open source) to segment, count and create a mask of PSD95^+^ puncta, and then count the number of receptor subunit puncta within this PSD95^+^ mask. Data are presented as the percentage of PSD95^+^ puncta containing receptor subunit puncta. The nuclear region was manually removed from CA1 images prior to analysis.

#### Golgi staining

To perform Golgi staining, animals were sacrificed, their brains were extracted, and the hemispheres were rinsed with double distilled water (ddH_2_O). The staining process was carried out based on the Rapid Golgi Staining Kit’s instructions from the manufacturer (FD NeuroTechnologies, USA). The brains were then impregnated and sliced into 100 μm thick sections using a cryotome (Thermo Fisher Scientific, USA). These sections were subsequently stained following the manufacturer’s instructions and embedded in Permount (Thermo Fisher Scientific, USA).

#### Image acquisition and analysis after Golgi staining

An Axio Observer Z1 inverted fluorescence microscope was used to obtain images of Golgi-stained neurons. Basal and mid-apical dendritic segments were imaged separately using a 63X oil objective and a z-step size of 130 nm. The exposure time for each frame was adjusted individually to use the full grayscale range. The data analysis was carried out following the same protocol as described previously,^12^ and the data acquisition and analysis conducted in a genotype-blind manner.

#### Single-nucleus RNA sequencing analysis of existing data

Single nucleus RNA sequencing data are from Zhou et al., 2020.^27^ Mouse raw data were downloaded from the Gene Expression Omnibus (GEO) database with accession number GSE140511, while human raw data were obtained via the AD Knowledge Portal (https://adknowledgeportal.org) study snRNAseqAD_TREM2. Data analysis and visualization were performed using R (4.1.2) and packages Seurat (4.0.6), DoubletFinder (2.0.3), dplyr (1.0.7), ggplot2 (3.3.5), pheatmap (1.0.12), and wesanderson (0.3.6). For the mouse data, we excluded nuclei with less than 300 or more than 5600 unique molecular identifiers (UMIs), nuclei with less than 300 or more than 9000 expressed genes, and nuclei with more than 5 % of UMI counts for mitochondrial-encoded genes. Next, we performed doublet analysis using DoubletFinder and retained only nuclei labeled as singlets. We then performed data normalization, variable gene identification, data scaling, dimensionality reduction with PCA and clustering analysis using functions implemented in the Seurat package. We labeled clusters according to the expression of a series of canonical markers for brain cell types, and we excluded clusters expressing markers of multiple cell types (Figure S1A, left). For the human data (Figure S1A right), we retained all cells included in the labeled metadata accompanying the study, without further quality control.

#### Computational modeling

To understand the differences between control and cTKO mice, we employed a mean-field theory of rate distributions in the balanced state, able to produce distributions ranging from log-normal like shapes up to distributions with large subpopulations of silent neurons^47^:

(Equation 1) $$\rho \left(\nu |\gamma ,\delta ,\nu^{max}\right)=\frac{\gamma }{\nu^{max}\sqrt{-\pi \;\ln\left(\frac{\nu }{\nu^{max}}\right)}}\;\exp\left(-\frac{\delta^{2}}{2}\right){\left(\frac{\nu }{\nu^{max}}\right)}^{\gamma^{2}-1}\cosh\;\left(\gamma \delta \sqrt{-2\;\ln\left(\frac{\nu }{\nu^{max}}\right)}\right)$$

with $\delta :=\frac{{\text{Ψ}}_{0}-\bar{l}}{\alpha }$, $\gamma :=\frac{\sigma_{V}}{\alpha }$.

Posterior distributions of parameters γ (*dark matter parameter*, ratio of temporal $\left(\sigma_{V}\right)$ and quenched $\left(\alpha \right)$ standard deviation of membrane potential and input current, respectively), δ (relative distance of average input current $\bar{I}$ toward firing threshold $\Psi_{0}$), and $\nu^{max}$ (model maximum rate, determined by autocorrelations in membrane potential fluctuations) were obtained from empirical transient count distributions of Ca^2+^-imaging using nested sampling.^94,95^ Posterior distributions for control and cTKO mice revealed a largely uninformative posterior along $\nu^{max}$ with no significant difference between cTKO and control mice (see Figure S4A).

Using a Gauss-Rice neuron model, a leaky integrate-and-fire (LIF)-model without reset, reproducing LIF-like behavior in the regime of low firing rates, the network model describes the distribution of neuron activity rates in a balanced state. Activity events per neuron are counted as upward threshold crossings of its membrane potential, ignoring intrinsic mechanisms of burst generation and merely imposing Poisson-like dynamics, as observed in *in vivo* data.^96^ This allows its application to calcium transient counts in which the actual number of emitted action potentials is linked to the transient intensity through an unknown function.

The solutions for rate distributions across the network (Equation 1) allow for a singularity at ν→0, resulting in a large subpopulation of neurons active at arbitrarily low rates. However, the empirical data is present in the form of calcium transient counts from a finite measurement period. We assume the observed counts to be a realization of a Poisson Point Process with the underlying transient rate drawn from the theoretically obtained rate distribution. According to the Poisson distribution, the probability of a neuron with rate ν to exhibit $N_{TC}$ transients in time T is given by:

$$p\left(N_{TC}|\nu ,\;T\right)=\frac{{\left(\nu T\right)}^{N_{TC}}}{N_{TC}!}e^{-\nu T}$$

Thus, the probability of any one neuron within the population to exhibit $N_{TC}$ transients is:

$$p_{\text{\#}}=p\left(N_{TC}|\text{Σ},\;T\right)=\int_{0}^{\nu^{max}}\rho \left(\nu |\text{Σ}\right)p\left(N_{TC}|\nu ,T\right)\text{d}\nu$$

where Σ specifies the set of parameters $\left(\gamma ,\;\delta ,\;\nu^{max}\right)$. The probability of observing $k\left(N_{TC}|\text{Σ},\;n,\;T\right)$ neurons with transient count $N_{TC}$ over time T within a population of n recorded neurons is obtained from the binomial distribution, where the dependencies are dropped for readability:

$$p\left(k|\text{Σ},n,N_{TC},T\right)=\left(\begin{array}{c} n\\ k \end{array}\right)p_{\text{\#}}^{k}{\left(1-p_{\text{\#}}\right)}^{n-k}$$

This expression serves as a likelihood to observe k neurons with transient count $N_{TC}$. The overall log likelihood evaluates to:

$$\log\;p_{\text{data}}=\sum_{N_{TC}}\text{log}\;p\left(k|\text{Σ},n,\;N_{TC},\;T\right)$$

which is then used to sample the parameter space of Σ with the nested sampling^95^ Monte Carlo algorithm MLFriends^97,98^ using the UltraNest package.^94^

#### Inferring the fraction of NMDARs

In our model, we describe interactions between neurons by the postsynaptic kernel, defined for the excitatory population as:

(Equation 2) $$f_{I}\left(t\right)=\frac{1}{\tau_{G}}e^{-\frac{1}{\tau_{G}}}\text{Θ}\left(t\right),\;f_{E}\left(t\right)=\frac{1-r}{\tau_{A}}e^{-\frac{1}{\tau_{A}}}\text{Θ}\left(t\right)+\frac{r}{\tau_{N}}e^{-\frac{1}{\tau_{N}}}\text{Θ}\left(t\right)$$

with r being the ratio of NMDA (here $\tau_{N}=150\;\text{ms}$ ^99^) vs. AMPA (here $\tau_{A}=1\;\text{ms}$ ^99^) receptors in excitatory neurons. The model allows combining multiple populations with either inhibitory or excitatory impact, requiring at least one non-quiescent inhibitory population, to avoid exploding solutions (diverging firing rates). Inhibitory postsynaptic currents are described by an according kernel with a single time constant for GABA (here $\tau_{G}=5\;\text{ms}$ ^100^), only. Theory then defines $\nu^{max}$ as:

(Equation 3) $$\nu^{max}=\frac{1}{2\pi }\frac{\sigma_{\dot{V}}}{\sigma_{V}},\;\sigma_{V}^{2}=\sum_{l}\sigma_{V_{l}}^{2},\;\sigma_{\dot{V}}^{2}=\sum_{l}\sigma_{{\dot{V}}_{l}}^{2}$$

With,

Excitatory synapses: $\sigma_{V_{A}}^{2}=\frac{J^{2}\kappa_{E}{\bar{\nu }}_{E}}{\tau_{A}+\tau_{M}}\left[\frac{{\left(1-r\right)}^{2}}{2}+\frac{\left(1-r\right)r\tau_{A}}{\tau_{A}+\tau_{N}}\right]$, $\sigma_{V_{N}}^{2}=\frac{J^{2}\kappa_{E}{\bar{\nu }}_{E}}{\tau_{N}+\tau_{M}}\left[\frac{r^{2}}{2}+\frac{\left(1-r\right)r\tau_{N}}{\tau_{A}+\tau_{N}}\right]$

Inhibitory synapses: $\sigma_{V_{G}}^{2}=\frac{J^{2}{\bar{\nu }}_{l}}{2\left(\tau_{G}+\tau_{M}\right)}$

$$\sigma_{{\dot{V}}_{j}}^{2}=\frac{\sigma_{V_{j}}^{2}}{\tau_{j}\tau_{M}}$$

with J the synaptic weight (chosen to be same for all connections) and $\kappa_{E}$ the relative size of the excitatory population toward the inhibitory one (≈4, see^101^). $\bar{\nu }$ is the excitatory/inhibitory population mean firing rate and $\tau_{M}\left(\text{=}10\;\text{ms}\right)$ the membrane time constant. The value of $\nu^{max}$ describes the fastest timescale at which the membrane potential of a neuron can cross the threshold from below, turn around twice, and cross it again from below. It is a highly theoretical value, as it neglects hyperpolarization after an action potential has been emitted, and thus assumes the membrane potential to fluctuate around the firing threshold $\Psi_{0}$. The model loses plausibility and comparability to more realistic neuron models in this regime, and is thus applicable at lower rates, only. The value of $\nu^{max}$, however, remains important for the description of the rate distributions, as it defines its scale and is important for the link toward the underlying parameters.

As the posterior remained uninformative and unchanged across mouse lines, we assumed $\nu^{max}$ to be constant and added an inhibitory population active at about twice the excitatory rate observed (here ≈ 4 min^−1^, assumed, as no data available) to allow for a balanced state. With ${\bar{\nu }}_{E}$ provided by the empirical data (see also^47^), the NMDA ratio $r_{NMDA}$ was obtained from Equation 3. The exact value depends on the chosen value of $\nu^{max}$ (here chosen to be 30 s^−1^ such that control mice have a sensible ratio of NMDA receptors $r_{NMDA}$, but the qualitative effect of reduced ${\text{r}}_{NMDA}$ in cTKO mice is robust against changes in the chosen parameters.

#### Power spectral density

We calculate the power spectral density of a membrane potential $V\left(t\right)$ in response to a mixed kernel of AMPA and NMDA transmitters:

$$V\left(t\right)=\int_{-\infty }^{\infty }G\left(t-s\right)f\left(s\right)\text{d}s,\;G\left(t\right):=\frac{1}{\tau_{M}}e^{-\frac{t}{\tau_{M}}}\text{Θ}\left(t\right)$$

with G the Greens function of the membrane potential response to arbitrary incoming currents. The overall response to a single action potential $f\left(t\right)$ of shape Equation 2 is thus:

$$V\left(t\right)=\frac{1}{\tau_{M}}\int_{-\infty }^{\infty }e^{-\frac{t-s}{\tau_{M}}}\text{Θ}\left(t-s\right)\left[\frac{1-r}{\tau_{A}}e^{-\frac{s}{\tau_{A}}}+\frac{r}{\tau_{N}}e^{-\frac{s}{\tau_{N}}}\right]\text{Θ}\left(s\right)\text{d}s$$

$$=\frac{1-r}{\tau_{A}-\tau_{M}}e^{-\frac{t}{\tau_{A}}}+\frac{r}{\tau_{N}-\tau_{M}}e^{-\frac{t}{\tau_{N}}}-\left(\frac{1-r}{\tau_{A}-\tau_{M}}+\frac{r}{\tau_{N}-\tau_{M}}\right)e^{-\frac{t}{\tau_{M}}}\text{Θ}\left(t\right)$$

The Fourier Transform of this expression evaluates to:

$$FT\left[V\left(t\right)\right]=\hat{V}\left(\omega \right)=\frac{1}{\sqrt{2\pi }}\int_{-\infty }^{\infty }V\left(t\right)e^{-i\omega t}\text{d}t$$

$$=\frac{1}{\sqrt{2\pi }}\left[\frac{\tau_{A}}{\tau_{A}-\tau_{M}}\frac{\left(1-r\right)\left(1-i\tau_{A}\omega \right)}{1+\tau_{A}^{2}\omega^{2}}+\frac{\tau_{N}}{\tau_{N}-\tau_{M}}\frac{r\left(1-i\tau_{N}\omega \right)}{1+\tau_{N}^{2}\omega^{2}}-\left[\frac{\left(1-r\right)\tau_{M}}{\tau_{A}-\tau_{M}}+\frac{r\tau_{M}}{\tau_{N}-\tau_{M}}\right]\frac{1-i\tau_{M}\omega }{1+\tau_{M}^{2}\omega^{2}}\right]$$

Finally, the power spectral density is defined as:

SVV(ω)=|V^ω|2=ReV^ω2+ImV^ω2

which is displayed in Figure 4B for different values of $r_{NMDA}$. The band power between $\omega_{a}$ and $\omega_{b}$ is defined as:

$$\int_{\omega_{a}}^{\omega_{b}}S_{VV}\left(\omega \right)\text{d}\omega .$$

#### Behavioral experiments and analysis

Behavioral experiments and data presented in Figure 7 were conducted in accordance with the Animal (Scientific Procedures) Act 1986, approved by the Animal Welfare and Ethical Review Body (AWERB) at UCL, and performed under an approved UK Home Office project license at UCL. Open field tests were conducted in a standard lit room during the light-phase, with a 1 m circular white Perspex arena. Locomotion in the arena was recorded using a GoPro Hero4 camera mounted to an overhead gantry and animal movement tracked using custom written MATLAB scripts. 24 h before testing, animals were habituated to the room for 1 h whilst in their cages. On the day of the experiment, animals were allowed to settle for a further 1 h after transport and before testing. Testing followed a crossover design and spanned two consecutive weekends. For the first day, 6 animals (3 littermate controls, 3 cTKOs) were randomly allocated (using custom MATLAB scripts) to either saline (as a vehicle control) or D-cycloserine (DCS, 30 mg/kg) i.p. injection, and subsequently underwent the alternative 7 days later. On the second day, an additional 6 animals underwent the same protocol, yielding a total of 12 animals (6 × littermate controls, 6 × cTKOs) that had undergone both DCS treatment and saline injection. The independent experimenter was blinded to genotype and the treatment being administered (test schedule and pre-labelled solution vials bearing only the animal identification number). In light of functional data suggesting beneficial effects ∼20 min following DCS administration, mice were injected 10 min prior to placement in the center of the open field and locomotor movement was recorded for a subsequent 30 min. Locomotion paths over time for each animal (30 Hz) were first downsampled to 3 Hz to improve computational time (MATLAB function ‘decimate’, employing a finite impulse response filter with a Hamming window and filter order 30). Total distance covered was then binned into 3 × 10 min epochs. In order to capture potential dynamic effects of DCS on animal locomotion and stereotypic behavior, we sub-divided the arena into 9 × 9 numbered square tiles, and locomotion paths of animals were analyzed for repeating movements between tiles over the 30 min recording period. Each tile visit repetition increased the stereotypy count of all involved tiles, and stereotypy index was defined as the total stereotypy counts divided by total number of tile crossings, and normalized to the saline condition in littermate controls.

### QUANTIFICATION AND STATISTICAL ANALYSIS

Sample sizes were chosen using standards in the field and established in previously published studies. Statistical analyses were conducted in MATLAB. We leveraged a number of linear mixed-effects (LME) models to model a variety of fixed (e.g., behavioral covariates) effects and factors (e.g., neuronal activity class) and random effects (e.g., inter-subject variability and the nested nature of experimental sessions) as appropriate, with general model design described in the main text and model-derived coefficient estimates and/or *p*-values presented in figure legends where relevant. LME models were compared using MATLAB function “compare”, and conditional predictions from LME models (incorporating contributions from both fixed and random effects) with 95 % confidence intervals generated using MATLAB function “predict”. Model residuals were visually confirmed for an approximate normal distribution by computation of skewness, kurtosis and fitting of a normal density function (MATLAB function “histfit”). LME model formulas and outputs for main figures are described in detail in Tables S1–S7. Tests for normality were performed to inform the subsequent use of standard parametric or non-parametric statistical tests, including pairwise and paired comparisons, and repeated measures and 1-way ANOVAs, with Tukey-Kramer correction for multiple comparisons, where necessary. Results were considered significant at *p* < 0.05. Statistical tests and the nature and number of biological units, definition of center, dispersion and precision measures, as appropriate, are detailed in the associated figure legends.

## Supplementary Material

1

Supplemental information can be found online at https://doi.org/10.1016/j.celrep.2025.115801.

## Figures and Tables

**Table:** KEY RESOURCES TABLE

REAGENT or RESOURCE	SOURCE	IDENTIFIER
Antibodies		
PSD95	Abcam	Cat# ab2723; RRID:AB_303248
GluN1	Synaptic Systems	Cat# 114 103; RRID:AB_2112011
GluA1	Abcam	Cat# ab31232; RRID:AB_2113447
GluA2	Synaptic Systems	Cat# 182 105; RRID:AB_2619875
Goat anti-Mouse Alexa Fluor 488	Invitrogen	Cat# A11001; RRID:AB_2534069
Goat anti-Mouse Alexa Fluor 647	Invitrogen	Cat# A21235; RRID:AB_2535804
Goat anti-Rabbit Alexa Fluor 594	Invitrogen	Cat# A11012; RRID:AB_2534079
Goat anti-Guinea Pig Alexa Fluor 488	Invitrogen	Cat# A11073; RRID:AB_2534117
Bacterial and virus strains		
pGP-AAV-*syn*-jGCaMP8s-WPRE (AAV1)	pGP-AAV-*syn*-jGCaMP8s-WPRE was a gift from GENIE Project. Zhang et al.^74^	Addgene viral prep # 162374-AAV1; http://n2t.net/addgene:162374; RRID:Addgene_162374
pAAV.Syn.NES-jRCaMP1b.WPRE.SV40 (AAV1)	pAAV.Syn.NES-jRCaMP1b. WPRE.SV40 was a gift from Douglas Kim & GENIE Project. Dana et al.^75^	Addgene viral prep # 100851-AAV1; http://n2t.net/addgene:100851; RRID:Addgene_100851
pAAV.Syn.GCaMP6f.WPRE.SV40 (AAV1)	pAAV.Syn.GCaMP6f.WPRE.SV40 was a gift from Douglas Kim & GENIE Project. Chen et al.^76^	Addgene viral prep # 100837-AAV1; http://n2t.net/addgene:100837; RRID:Addgene_100837
Chemicals, peptides, and recombinant proteins		
Dichloromethane	Sigma-Aldrich	Cat# 270997
Benzyl ether	Sigma-Aldrich	Cat# 108014
Vybrant CM-DiI Cell-Labeling Solution	ThermoFisher	Cat# V22888
MK 801	Merck	Cat# M107
D-Cycloserine	Cambridge Bioscience	Cat# HY-B0030R
CX546	Tocris	Cat# 2980
Critical commercial assays		
FD Rapid GolgiStain kit	FD Neurotechniques, USA	Cat# PK401
Deposited data		
Source Data to reproduce key quantitative comparisons	This Paper	Zenodo (https://doi.org/10.5281/zenodo.15210928)
Experimental models: Organisms/strains		
Mouse: NexCre-cTKO: B6.129-*NeuroD6*^*tm1(cre)Kan*^ *App*^*tm2UhG*^*Aplp1*^*tm1*^*Aplp2*^*tm2UhG*^/UhG	Bred in house (Müller Lab). Steubler et al.^12^	N/A
Mouse: Control littermate: B6.129-*App*^*tm2UhG*^ *Aplp1*^*tm1*^*Aplp2*^*tm2UhG*^/UhG	Bred in house (Müller Lab). Steubler et al.^12^	N/A
Mouse: APLP1-KO: B6.129-*Aplp1*^*tm1*^/UhG	Bred in house (Müller Lab) Heber et al.^14^; Erdinger et al.^30^	N/A
Mouse: APLP2-KO: B6.129-*Aplp2*^*tm1*^/UhG	Originally obtained from S. Sisodia, bred in house (Müller Lab). Same line as deposited in JAX: 004142. Heber et al.^14^	JAX: 004142
Mouse: APP-KO: B6.129-*App*^*tm1*^/UhG	Bred in house (Müller Lab). Li et al.^77^	N/A
Mouse: WT: C57BL/6J	Charles River	https://www.criver.com/products-services/find-model/jax-c57bl6j-mice?region=23
Mouse: BTBR: BTBR T + Itpr3tf/J	JAX	JAX: 002282
Mouse: WT: C57BL/6	Bred in house (Busche Lab)	C57BL/6J (CRL)
Software and algorithms		
Neurolucida and Explorer	MBF Bioscience	https://www.mbfbioscience.com/products/neurolucida/
Cascade	GitHub (Rupprecht et al.^32^)	github.com/HelmchenLabSoftware/Cascade
Suite2p	GitHub (Pachitariu et al.^78^)	github.com/MouseLand/suite2p
Cellpose	GitHub (Stringer et al.^79^)	github.com/MouseLand/cellpose
MATLAB 2024A	The MathWorks	https://se.mathworks.com/products/matlab.html
ImageJ	Schneider et al.^80^	https://imagej.nih.gov/ij/
SpikeGLX 3.0	GitHub	https://billkarsh.github.io/SpikeGLX/
Igor Pro 5	WaveMetrics	https://www.wavemetrics.com/software
Kilosort 3	GitHub (Pachitariu et al.^81^)	https://github.com/MouseLand/Kilosort (Version 4 now provided)
PHY	GitHub	https://github.com/cortex-lab/phy
Neuropixels Trajectory Explorer	GitHub	https://github.com/petersaj/neuropixels_trajectory_explorer
Facemap	GitHub (Syeda et al.^82^)	https://github.com/MouseLand/facemap
MATLAB scripts for visualization and statistical analyses of key source data	This paper	Zenodo (https://doi.org/10.5281/zenodo.15210936)
R Project for Statistical Computing (4.1.2)	The R Foundation	http://www.r-project.org/
Seurat 4.0.6	GitHub	https://github.com/satijalab/seurat
DoubletFinder (2.0.3)	GitHub	https://github.com/chris-mcginnisucsf/DoubletFinder
dplyr (1.0.7)	GitHub	https://github.com/tidyverse/dplyr
ggplot2 (3.3.5)	GitHub	https://github.com/tidyverse/ggplot2
pheatmap (1.0.12)	GitHub	https://github.com/raivokolde/pheatmap
wesanderson (0.3.6)	GitHub	https://github.com/karthik/wesanderson
Chronux 2.11	Bokil et al.^83^	https://chronux.org/
Other		
Neuropixels 1.0 probes	IMEC	PRB_1_4_0480_1_C
KwikCast	World Precision Instruments	N/A
Dental Cement	Lang Dental	Cat#1223CLR
Super-Bond C&B	Prestige Dental	N/A
KwikCast	World Precision Instruments	N/A

## Highlights

- Conditional triple KO (cTKO) of APP family suppresses cortico-hippocampal activity
- Dysfunction is coupled to synaptic GluN1 (NMDAR) downregulation
- Experimental and computational models link NMDARs to spontaneous neuronal activity
- Upregulating NMDARs in cTKOs rectifies impaired neuronal dynamics and behavior
