# Supplementary material for: The amyloid precursor family of proteins in excitatory neurons are essential for regulating cortico-hippocampal circuit dynamics in vivo
Source: Cell Rep. Author manuscript; Available in PMC 2025 Jul 30. (PMC12309856; doi:10.1016/j.celrep.2025.115801)
Supplement: 1 [file NIHMS2092689-supplement-1.pdf]

Cell Reports, Volume *44*

Supplemental information

The amyloid precursor family of proteins in excitatory neurons are essential for regulating cortico-hippocampal circuit dynamics *in vivo*

Samuel S. Harris, Rikesh M. Rajani, Jana Zünkler, Robert Ellingford, Mengke Yang, James M. Rowland, Alexander Schmidt, Byung Il Lee, Marten Kehring, Mariam Hellmuth, Francesca Kar Wey Lam, Dominique Fässler, Susanne Erdinger, David P. Wolfer, Carlo Sala Frigerio, Fred Wolf, Bradley T. Hyman, Ulrike C. Müller, and Marc Aurel Busche

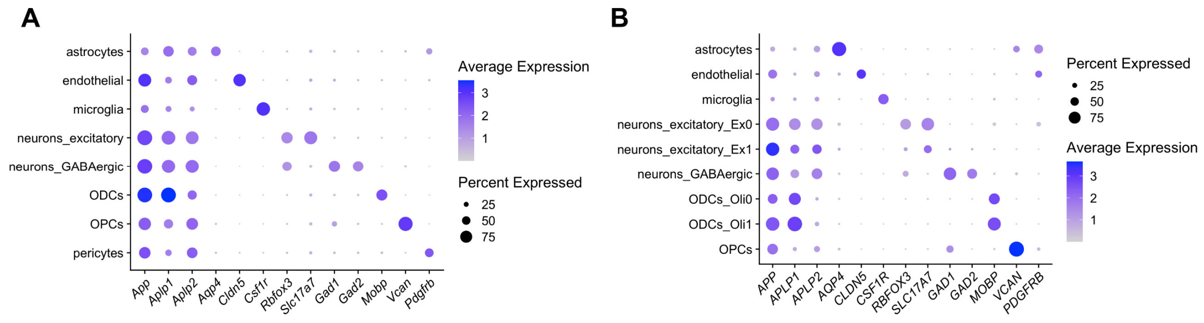

Figure S1: Pan-cellular expression of the APP family, related to STAR Methods. Pan-cellular expression of the APP family (**A**: mouse data; **B**: human data). Dot plots report the average expression of genes of interest (*App*, *Aplp1*, *Aplp2*) and of selected cell type markers for each of the cell types identified. The size of the dot is proportional to the percentage of cells of each type expressing the gene, and the colour is proportional to the level of normalized expression. ODCs: oligodendrocytes, OPCs: oligodendrocyte precursor cells. Data from Zhou et al. (2020).

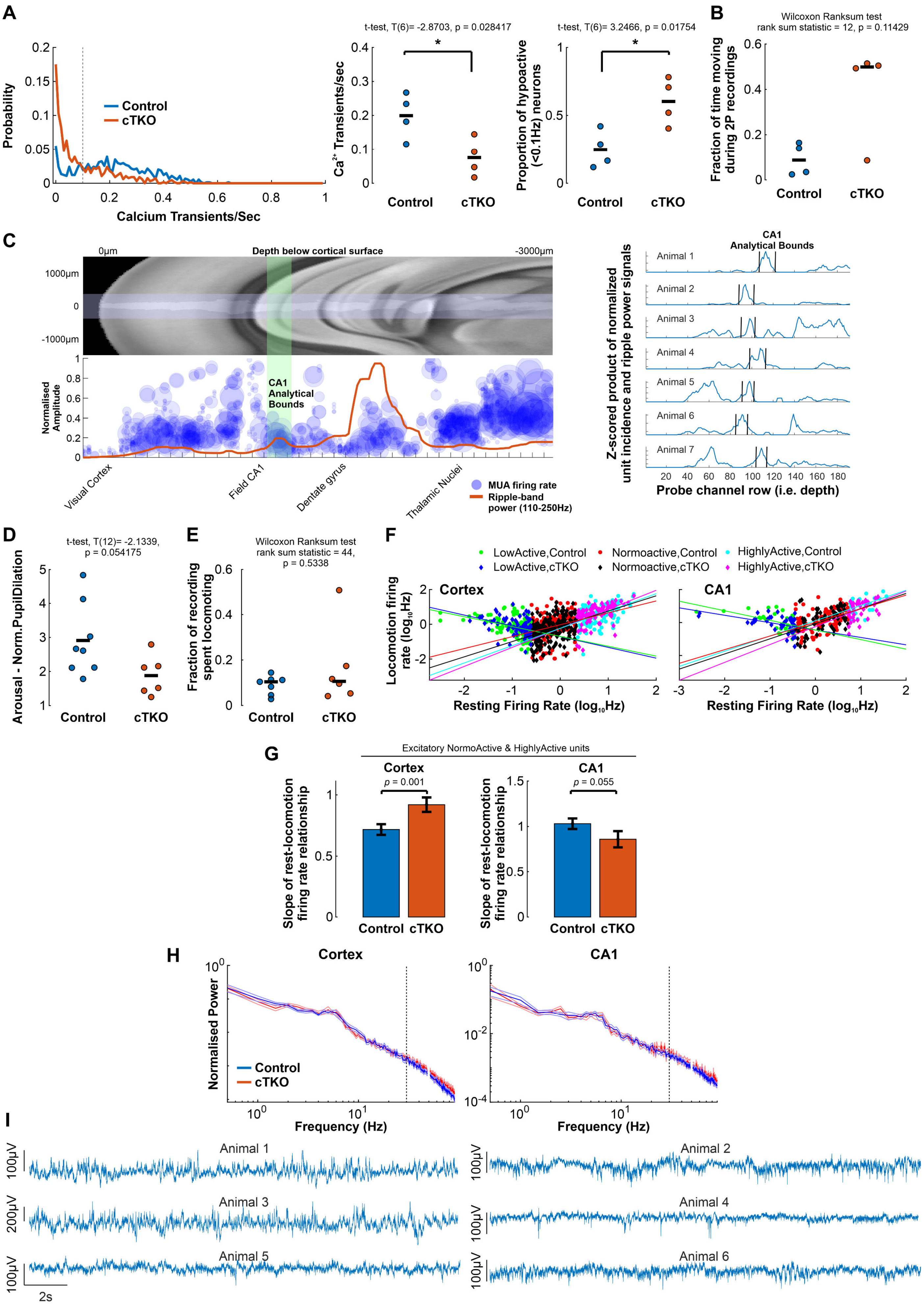

A
Probability
Control
cTKO
Calcium Transients/Sec
t-test, T(6)= -2.8703, p = 0.028417
Ca2+ Transients/sec
Control
cTKO
t-test, T(6)= 3.2466, p = 0.01754
Proportion of hypoactive (<0.1Hz) neurons
Control
cTKO
B
Wilcoxon Ranksum test
rank sum statistic = 12, p = 0.11429
Fraction of time moving during 2P recordings
Control
cTKO
C
0µm
Depth below cortical surface
-3000µm
1000µm
-1000µm
Normalised Amplitude
CA1 Analytical Bounds
Visual Cortex
Field CA1
Dentate gyrus
Thalamic Nuclei
MUA firing rate
Ripple-band power (110-250Hz)
Z-scored product of normalized unit incidence and ripple power signals
CA1 Analytical Bounds
Animal 1
Animal 2
Animal 3
Animal 4
Animal 5
Animal 6
Animal 7
Probe channel row (i.e. depth)
D
t-test, T(12)= -2.1339, p = 0.054175
Arousal - Norm.PupilDilation
Control
cTKO
E
Wilcoxon Ranksum test
rank sum statistic = 44, p = 0.5338
Fraction of recording spent locomoting
Control
cTKO
F
LowActive,Control
Normoactive,Control
HighlyActive,Control
LowActive,cTKO
Normoactive,cTKO
HighlyActive,cTKO
Cortex
CA1
Locomotion firing rate (log10Hz)
Resting Firing Rate (log10Hz)
Resting Firing Rate (log10Hz)
G
Excitatory NormoActive & HighlyActive units
Cortex
p = 0.001
CA1
p = 0.055
Slope of rest-locomotion firing rate relationship
Control
cTKO
Control
cTKO
H
Cortex
CA1
Normalised Power
Control
cTKO
Frequency (Hz)
Frequency (Hz)
I
Animal 1
Animal 2
Animal 3
Animal 4
Animal 5
Animal 6
100µV
200µV
2s

Figure S2: Awake 2-photon calcium imaging and Neuropixels, related to Figures 1-2 and STAR Methods. (**A**) Calcium transient rates in cTKO mice were significantly reduced versus controls with an increase in the fraction of low-activity neurons (each data-point represents an individual animal, N=4 controls, and N=4 cTKO mice). (**B**) Non-significant increase in fraction of time moving for cTKO mice versus controls during awake 2P recordings. Each data-point represents an individual animal. (**C**) Left: Neuropixels probe trajectory estimation was validated using mesoSPIM lightsheet imaging. Top, Compound image of lightsheet data, comprising of DiI labelled Neuropixels probe track (highlighted by pale blue horizontal band), overlaid with spatially registered Allen CCF v3 atlas image (image rotated to horizontal for visualization) in an example animal. Bottom, multi-unit activity (MUA) along the probe shank in the same animal (blue circles represent single units with radii indicating normalised firing rate and normalized unit amplitude on y-axis), together with power in the ripple band (110-250Hz, red) as a function of depth. Anatomical labels extracted from Allen CCF for same coronal slice shown on x-axis. The green vertical band encompassing the peak in ripple power within hippocampus CA1 denotes depth limits to which analysis for this brain region was restricted to. Right: Example traces in seven animals displaying the z-scored product of normalized unit incidence and ripple power as a function of depth (probe channels) and illustrating CA1 analytical bounds in each case. (**D**) Non-significant reduction in arousal (proxied by normalized pupil size) in cTKO animals compared to controls during awake Neuropixels recordings (N=8 controls, N=6 cTKOs). (**E**) No significant difference in fraction of time locomoting during awake Neuropixels recordings (N=7 controls, N=6 cTKOs, one control animal sessile during recordings). (**F**) Positive relationship between resting and locomotion firing rates (as log10Hz, silent neurons excluded) for normoactive and highly active excitatory neurons, but a negative relationship for low-active neurons, in both cortex and CA1 across genotypes (aggregated data from a single example recording session from each animal; Cortex: n=402 Control neurons from 7 animals, n=384 cTKO neurons from 6 animals; CA1: n=169 Control neurons from 7 animals, n=158 cTKO neurons from 6 animals). Lines indicate least squares regression fit for each condition. (**G**) Comparison of slopes derived from LME modelling for normoactive and highly active neurons, indicating a significant alteration in the relationship between resting and locomotion firing rates in cTKO mice versus controls in cortex (left), and a borderline significant effect in CA1 (right). Data represents LME derived estimates and associated standard errors. (**H**) Average LFP power spectra across sessions (normalized to area under the curve <250Hz) and animals for each genotype and brain region (error bars are SEM). Vertical dashed line indicates 30Hz frequency. (**I**) Example LFP traces from a randomly selected channel within CA1 analytical bounds in six animals.

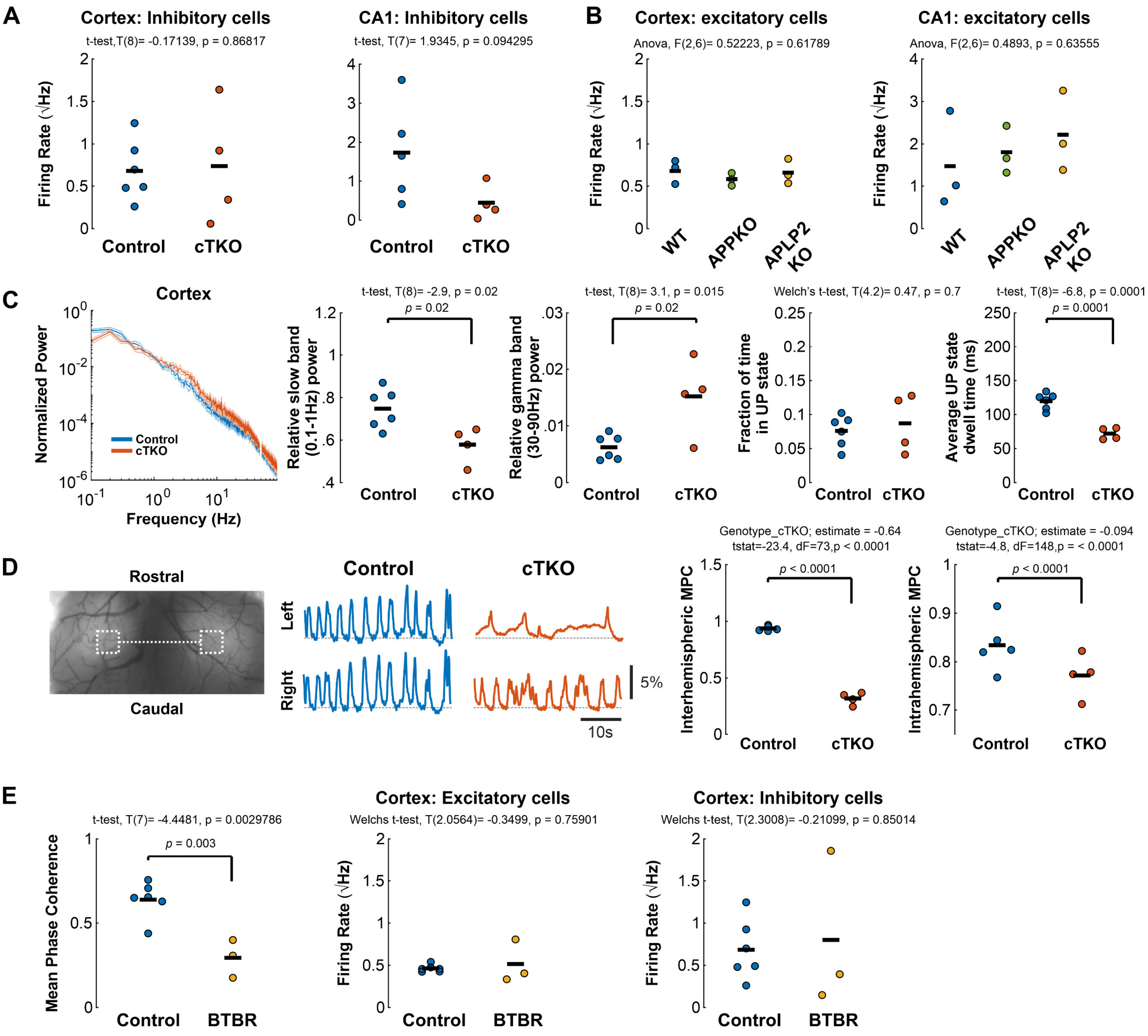

Figure S3: Additional controls and widefield calcium imaging during slow wave activity, related to Figures 3 and STAR Methods. (**A**) No significant difference in firing rate of putative inhibitory neurons in cTKO mice versus controls in cortex or CA1 (each datapoint represents an individual animal, N=6/5 controls, N=4 cTKOs). (**B**) No significant difference in firing rate of excitatory neurons across WT, APP KO and APLP1 KO mice in cortex and CA1 (each datapoint represents an individual animal, N=3 for each genotype). (**C**) Cortical LFP power spectra averaged across animals for each genotype (left, error bars are SEM), with a significant reduction in relative slow-wave band power and a concurrent increase in gamma-band power (left). While there was no significant difference between genotypes in the fraction of time spent in an UP state, UP state transitions were significantly shorter in cTKOs compared to controls (right). Each datapoint represents an individual animal, N=6 controls, N=4 cTKOs. (**D**) Widefield GCaMP6f calcium fluorescence imaging of superficial cortex during SWA (example image shown in left panel) revealed altered slow wave mean phase coherence (MPC) between hemispheres (example traces from ROIs in middle panel, with quantification in right panels, N=36 control traces and 39 cTKO traces across 5 and 4 mice per genotype, respectively) and within hemispheres (N=72 control traces and 78 cTKO traces across 5 and 4 mice per genotype, respectively). *p* values and coefficient estimates from LME models as insets. (**E**) Significant reduction in cross hemispheric cortical mean phase coherence (MPC) in BTBR mice lacking a corpus callosum, relative to control animals, despite comparable neuronal firing rates in cortical excitatory and inhibitory populations at the animal level. Each datapoint represents an individual animal (N=6 control, N=3 BTBR).

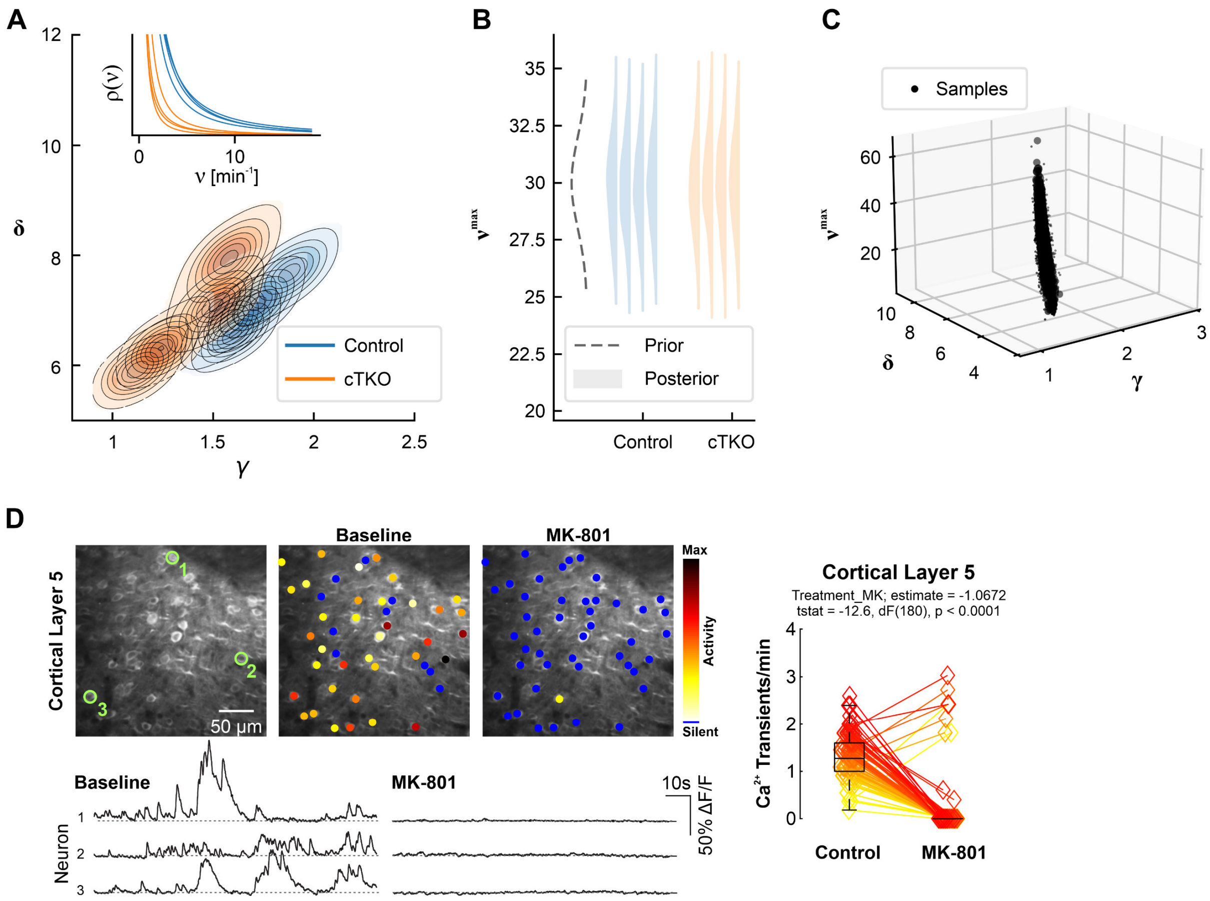

Figure S4: Additional computational modelling and impact of NMDAR antagonism on spontaneous neuronal activity in deep cortical layers, related to Figures 4-5 and STAR Methods. (**A**) Inference revealed that the inferred distributions (inset in a) occupy adjacent regions of parameter space along γ (dark matter parameter) and δ (relative distance of average input current towards firing threshold). (**B**) However, v^{max} (model maximum rate, determined by autocorrelations in membrane potential fluctuations) was largely uninformative as it reproduced arbitrary priors and showed no differences between control and cTKO mice, and was thus assumed to be constant. (**C**) The differences in v^{max} are compensated for by marginal adjustments in γ and δ. (**D**) Top, example *in vivo* two-photon fluorescence images of jRCaMP1b-expressing layer 5 neurons in cortex of wild-type mice during baseline (no drug, top middle) and 45mins following administration of the NMDAR antagonist MK-801 (1mg/kg, i.p., top right). Coloured markers indicate mean levels of spontaneous Ca^{2+} activity in individual neurons with cool colors indicating hypoactivity. Bottom, spontaneous Ca^{2+} activity (dF/F) from three example neurons circled and numbered in top left panel, before (left) and after (right) MK-801 administration. Right, quantification of significant suppressant effect of MK-801 on spontaneous Ca^{2+} activity in layer 5 (N=91 neurons from two animals). *p* value and coefficient estimate from LME model as inset.

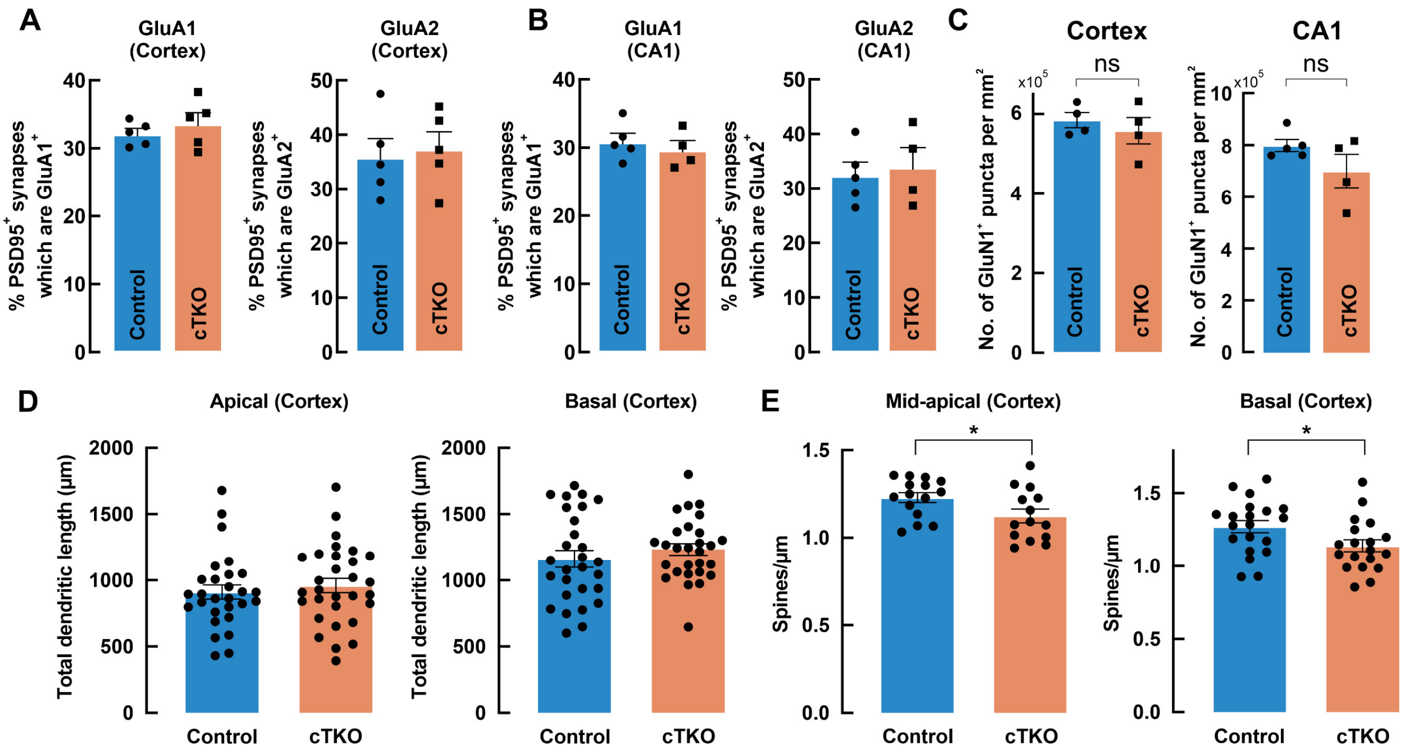

Figure S5: Additional immunohistochemical and histological characterisation of cTKO mice, related to Figure 6 and STAR Methods. (**A-B**) Quantification of immunofluorescence images of AMPA receptor subunits GluA1 and GluA2, in the cortex (retrosplenial and somatomotor, A) and medial CA1 (B) of controls and cTKO mice. Each datapoint represents an individual animal (minimum 4 FOV per animal, 4-5 mice per group), data as means with error bars as SEM; P≥0.05 in all cases with unpaired t-test. (**C**) No significant difference in number of GluN1-positive puncta per unit area between genotypes in cortex (left) or CA1 (right). Data as means with error bars as SEM; P≥0.05 in all cases with unpaired t-test (each datapoint represents an individual animal). (**D**) Left: Compared to controls, cortical layer 2/3 neurons of cTKO animals (N=28 control, 30 cTKO) showed no significant reduction in total dendritic length in apical (left) and basal (right) dendrites (P>0.05 unpaired t-test). (**E**) Left: There was a small but statistically significant reduction in spine density in mid-apical (left) and basal (right) dendritic segments of cortex layer 2/3 pyramidal neurons of cTKO animals compared to controls (left, N=15 control, 14 cTKO; right, N=20 control, 19 cTKO). D-E) Each datapoint represents an individual neuron, data as mean ± SEM; *P<0.05 unpaired t-test.

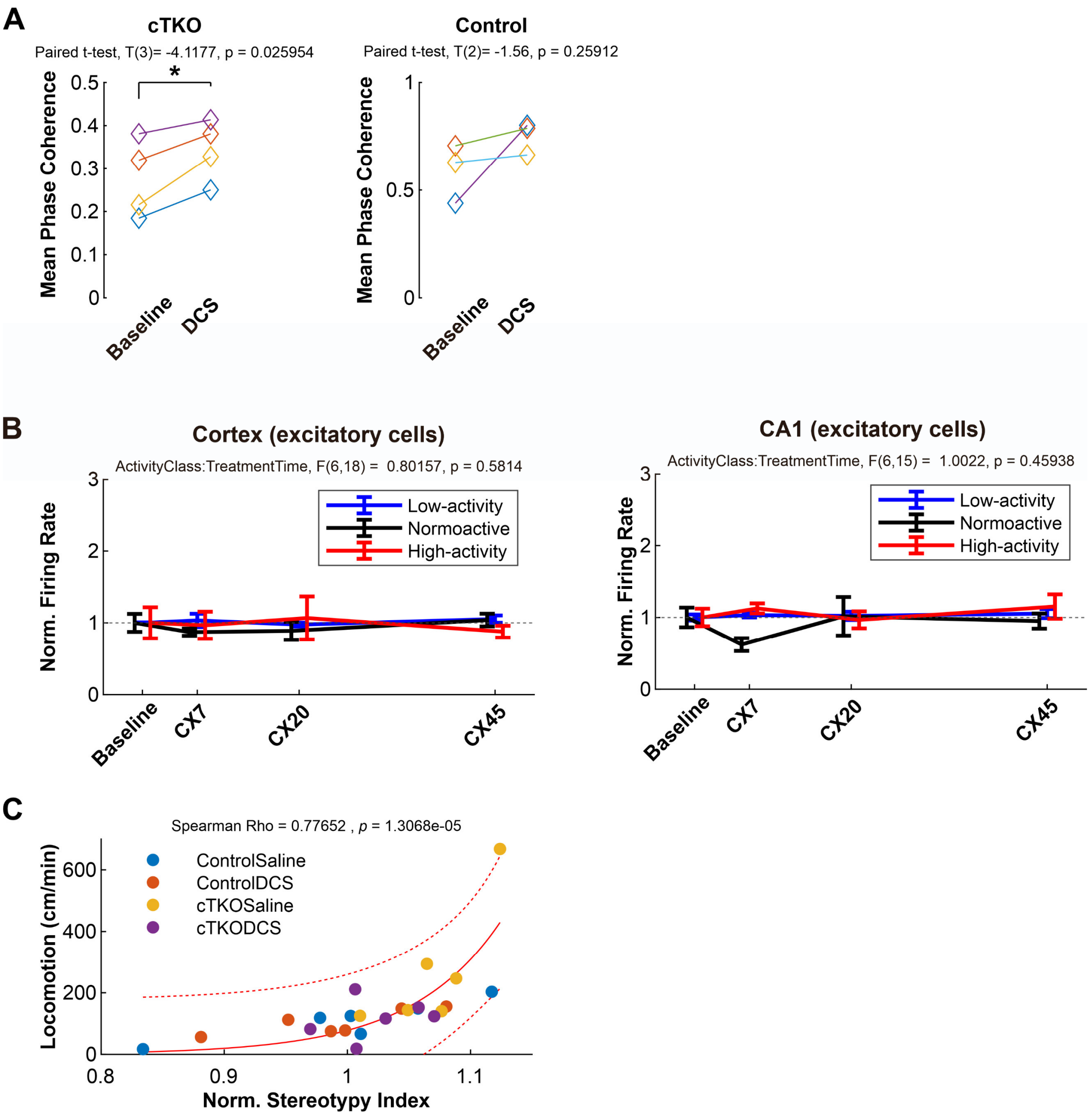

Figure S6: Extended characterization of the effects of NMDAR modulation on neuronal activity and behaviour, related to Figure 7 and STAR Methods. (A) Cross hemispheric cortical mean phase coherence was significantly increased by DCS treatment in cTKO animals (N=4, left) but not in control animals (N=3, right). (**B**) CX546 treatment (20mg/kg, i.p.) failed to induce significant changes in firing rate of different activity classes of excitatory neurons (classified according to baseline firing rates: low activity < 0.25Hz, normoactive >0.25 to < 4Hz, high activity > 4Hz) in cTKO animals (N=3, data represent animal averages with error bars as SEM, repeated measures ANOVA). (**C**) Significant monotonic relationship between locomotion and stereotypy in the open-field behavioral test, with no clear effect of genotype or DCS versus saline treatment.

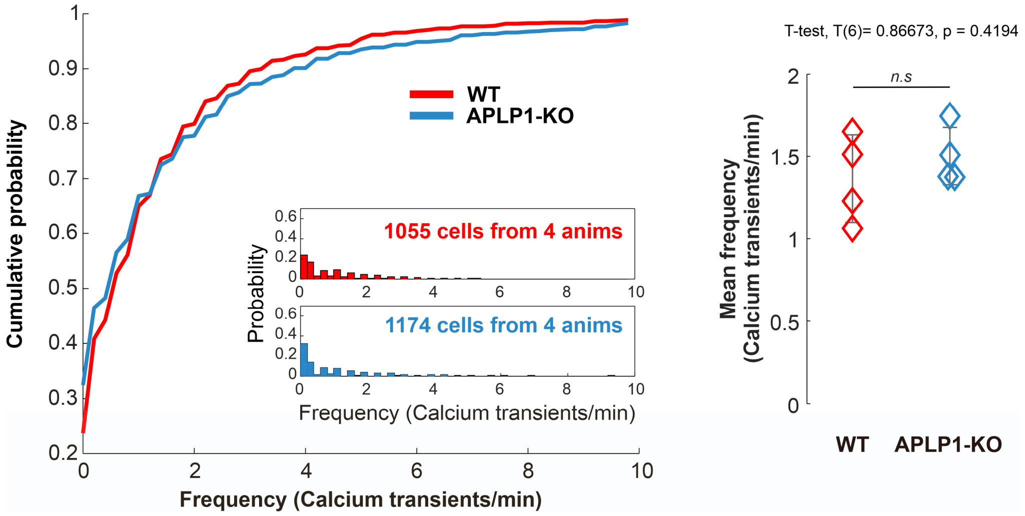

Figure S7: No difference in calcium transient rates in age-matched wild-types and internal controls, related to STAR Methods. Comparison of two-photon calcium imaging outcomes in age-matched wild-type (WT, N=4) and APLP1-KO mice (N=4, used as internal controls for cTKO mice in this study) during slow wave activity, and showing no significant difference in activity levels between genotypes (right, each datapoint represents an individual animal).

Table S1: Linear mixed effects modelling of genotype differences in resting neuronal firing rates obtained during awake 2-photon calcium imaging, related to Figure 1C.

FiringRate: Deconvolved neuronal firing rates.
MovCentered: mean subtracted fraction of time moving during recording.
Genotype: Control (reference level) or cTKO animals.
Data at single neuron level from N = 4 controls, 4 cTKO animals

LME Model: FiringRate ~ MovCentered * Genotype + (1|Animal_ID)

Name	Estimate	SE	tStat	DF	pValue	Lower	Upper
(Intercept)	1.290936665	0.111838664	11.54284766	1408	1.62972E-29	1.07154832	1.51032501
Genotype_cTKO	-0.559199449	0.126731763	-4.41246484	1408	1.09988E-05	-0.807802845	-0.310596052
MovCentered	0.897583446	0.694863748	1.291740212	1408	0.19665912	-0.465496207	2.260663099
Genotype_cTKO:MovCentered	-1.447432743	0.7374809	-1.9626715	1408	0.049881012	-2.894112343	-0.000753142

Table S2: Linear mixed effects modelling of genotype differences in resting neuronal firing rates across different classes obtained during awake Neuropixels recordings, related to Figure 2D.

RestFRClass: Neurons were categorised according to their resting state firing rate (RestFR) into 'LowActive' (<25th percentile), 'Normoactive' (25th-75th percentile, reference level) or 'HighlyActive' (>75th percentile) classes.
Genotype: Control (reference level) or cTKO animals.

LME Model: RestFR ~ RestFRClass*Genotype + (1|Animal_ID) + (1|Animal_ID:Session)

Putative Excitatory Neurons (Cortex) *Data at single neuron level from 8 Control and 6 cTKO animals*

Name	Estimate	SE	tStat	DF	pValue	Lower	Upper
(Intercept)	0.776654175	0.245794954	3.159764525	1929	0.001603449	0.294602455	1.258705896
Genotype_cTKO	-0.16098722	0.360060621	-0.447111432	1929	0.654844801	-0.867136142	0.545161702
RestFRClass_LowActive	-0.479245379	0.251427697	-1.906096209	1929	0.056786214	-0.972344004	0.013853246
RestFRClass_HighlyActive	7.526098798	0.26533588	28.36442168	1929	3.2023E-148	7.005723519	8.046474077
Genotype_cTKO:RestFRClass_LowActive	-0.030148628	0.376872024	-0.07999699	1929	0.936247948	-0.769267983	0.708970728
Genotype_cTKO:RestFRClass_HighlyActive	-1.042575639	0.362531661	-2.875819551	1929	0.004074029	-1.753570751	-0.331580526

Putative Excitatory Neurons (CA1) *Data at single neuron level from 8 Control and 6 cTKO animals*

Name	Estimate	SE	tStat	DF	pValue	Lower	Upper
(Intercept)	1.757871497	0.331959192	5.29544455	805	1.53298E-07	1.10626373	2.409479263
Genotype_cTKO	0.077839987	0.500093543	0.155650853	805	0.87634726	-0.903801263	1.059481236
RestFRClass_LowActive	-1.594888454	0.556427839	-2.866298812	805	0.00426134	-2.687109153	-0.502667754
RestFRClass_HighlyActive	11.73161869	0.533085851	22.00699696	805	2.11313E-84	10.68521634	12.77802105
Genotype_cTKO:RestFRClass_LowActive	-0.039039169	0.766201586	-0.050951564	805	0.959376757	-1.543027958	1.46494962
Genotype_cTKO:RestFRClass_HighlyActive	-2.534042638	0.764053222	-3.316578695	805	0.000952115	-4.03381437	-1.034270905

Putative Inhibitory Neurons (Cortex) *Data at single neuron level from 8 Control and 6 cTKO animals*

Name	Estimate	SE	tStat	DF	pValue	Lower	Upper
(Intercept)	1.329468848	0.266214398	4.993978005	234	1.15661E-06	0.804985582	1.853952113
Genotype_cTKO	-0.111218538	0.380567634	-0.292243817	234	0.770359157	-0.860995247	0.638558172
RestFRClass_LowActive	-1.211770405	0.402573577	-3.010059465	234	0.002898103	-2.004902203	-0.418638606
RestFRClass_HighlyActive	6.468034291	0.404110513	16.00560758	234	1.91785E-39	5.671874491	7.26419409
Genotype_cTKO:RestFRClass_LowActive	0.122348153	0.674713725	0.181333428	234	0.856262838	-1.206941551	1.451637857
Genotype_cTKO:RestFRClass_HighlyActive	-1.682635344	0.664371191	-2.532673551	234	0.011974665	-2.991548667	-0.373722022

Putative Inhibitory Neurons (CA1) *Data at single neuron level from 8 Control and 4 cTKO animals (insufficient number of interneurons in 2 cTKO mice)*

Name	Estimate	SE	tStat	DF	pValue	Lower	Upper
(Intercept)	3.95540796	1.401390251	2.822488566	70	0.006198697	1.160423235	6.750392685
Genotype_cTKO	-0.214642332	2.498542865	-0.085907004	70	0.931785593	-5.197828947	4.768544284
RestFRClass_LowActive	-2.484194255	1.089304096	-2.280533291	70	0.025624158	-4.656742771	-0.311645739
RestFRClass_HighlyActive	9.818448721	0.956014619	10.2701868	70	1.30377E-15	7.911737686	11.72515976
Genotype_cTKO:RestFRClass_LowActive	-0.654474449	2.083231793	-0.314163047	70	0.754331606	-4.809349249	3.500400352
Genotype_cTKO:RestFRClass_HighlyActive	-0.814058189	2.010263069	-0.404951074	70	0.686748349	-4.823401459	3.19528508

Table S3: Linear mixed effects modelling of genotype differences in relationship between locomotion speed and modulation index, related to Figure 2G.

RestFRClass: Neurons were categorized according to their resting state firing rate (RestFR) into 'LowActive' (<25th percentile), 'Normoactive' (25th-75th percentile, reference level) or 'HighlyActive' (>75th percentile) classes.
Genotype: Control (reference level) or cTKO animals.
Speed: Locomotion speed (raw rotary encoder measures in RPM)
Modulation Index = Locomotion induced changes from resting state, given by (LocFR-RestFR) / (LocFR+RestFR)

Putative Excitatory Neurons (Cortex) *Data at single neuron level from N = 7 control, 6 cTKO animals (one control animal sessile during recordings)*

LME Model 1: Modulation Index ~ RestFRClass * Genotype + (1|Animal_ID) + (1| Animal_ID:Session)
LME Model 2: Modulation Index ~ RestFRClass * Genotype * Speed + (1| Animal_ID) + (1| Animal_ID:Session) + (Speed| Animal_ID:Session)

Model	DF	AIC	BIC	LogLik	LRStat	deltaDF	pValue
Model 1	9	2737.6242	2787.394	-1359.81211			
Model 2	18	2716.0701	2815.609	-1340.03505	39.55	9	9E-06

Significant improvement in model fit for Model 2

LME Model: Modulation Index ~ RestFRClass * Genotype * Speed + (1|Animal_ID) + (1|Animal_ID:Session)

Name	Estimate	SE	tStat	DF	pValue	Lower	Upper
(Intercept)	0.021322302	0.162201782	0.131455411	1851	0.895429343	-0.296795363	0.339439967
Speed	0.002133293	0.028133042	0.075828741	1851	0.939563544	-0.053042535	0.057309122
Genotype_cTKO	-0.129004119	0.200424794	-0.643653497	1851	0.519879888	-0.522086529	0.26407829
RestFRClass_LowActive	-0.024273512	0.161219806	-0.150561599	1851	0.88033796	-0.34046528	0.291918256
RestFRClass_HighlyActive	-0.014186482	0.153357139	-0.092506171	1851	0.926305899	-0.314957622	0.286584658
Speed:Genotype_cTKO	0.000274774	0.028836314	0.009528748	1851	0.992398301	-0.056280344	0.056829892
Speed:RestFRClass_LowActive	0.090168105	0.026882671	3.354134847	1851	0.000812201	0.037444562	0.142891648
Speed:RestFRClass_HighlyActive	-0.037303092	0.025659036	-1.453799437	1851	0.146171444	-0.087626784	0.0130206
Genotype_cTKO:RestFRClass_LowActive	0.832677294	0.19094904	4.360730447	1851	1.36756E-05	0.458179171	1.207175417
Genotype_cTKO:RestFRClass_HighlyActive	-0.35599539	0.191374154	-1.860206214	1851	0.06301486	-0.731327265	0.019336485
Speed:Genotype_cTKO:RestFRClass_LowActive	-0.103371162	0.027555603	-3.751366402	1851	0.000181287	-0.157414489	-0.049327835
Speed:Genotype_cTKO:RestFRClass_HighlyActive	0.054349427	0.026331	2.064085149	1851	0.039148712	0.002707846	0.105991007

Putative Excitatory Neurons (CA1) *Data at single neuron level from N = 7 control, 6 cTKO animals (one control animal sessile during recordings)*

LME Model 1: Modulation Index ~ RestFRClass * Genotype + (1|Animal_ID) + (1| Animal_ID:Session)
LME Model 2: Modulation Index ~ RestFRClass * Genotype * Speed + (1| Animal_ID) + (1| Animal_ID:Session) + (Speed| Animal_ID:Session)

Model	DF	AIC	BIC	LogLik	LRStat	deltaDF	pValue
Model 1	9	1075.77651	1117.45266	-528.888256			
Model 2	18	1074.25552	1157.60782	-519.127758	19.52	9	0.021

Significant improvement in model fit for Model 2

LME Model: Modulation Index ~ RestFRClass * Genotype * Speed + (1|Animal_ID) + (1|Animal_ID:Session)

Name	Estimate	SE	tStat	DF	pValue	Lower	Upper
(Intercept)	-0.049902679	0.176661914	-0.282475594	746	0.777657211	-0.396716347	0.296910989
Speed	-0.009405067	0.0301102	-0.312354864	746	0.754858137	-0.068515878	0.049705743
Genotype_cTKO	-0.319563617	0.230091717	-1.388853196	746	0.165291875	-0.771267953	0.132140719
RestFRClass_LowActive	0.1642789	0.248609594	0.660790667	746	0.50895057	-0.323778789	0.652336589
RestFRClass_HighlyActive	-0.057414626	0.254971103	-0.225180913	746	0.821900207	-0.557960905	0.443131654
Speed:Genotype_cTKO	0.034452844	0.031523835	1.092914136	746	0.274784301	-0.027433141	0.09633883
Speed:RestFRClass_LowActive	0.054363788	0.042453522	1.280548362	746	0.200750341	-0.028978803	0.13770638
Speed:RestFRClass_HighlyActive	0.034546259	0.043066825	0.802154767	746	0.42271889	-0.050000337	0.119092855
Genotype_cTKO:RestFRClass_LowActive	0.886854209	0.280466421	3.162069119	746	0.001629939	0.336256821	1.437451597
Genotype_cTKO:RestFRClass_HighlyActive	0.008562825	0.288402372	0.029690549	746	0.976321791	-0.557614018	0.574739667
Speed:Genotype_cTKO:RestFRClass_LowActive	-0.09065148	0.043505104	-2.083697573	746	0.037527511	-0.176058485	-0.005244476
Speed:Genotype_cTKO:RestFRClass_HighlyActive	-0.03872331	0.044223327	-0.875630873	746	0.381512579	-0.125540293	0.048093672

Putative Inhibitory Neurons (Cortex) *Data at single neuron level from N = 7 control, 6 cTKO animals (one control animal sessile during recordings)*

LME Model: Modulation Index ~ RestFRClass * Genotype * Speed + (1|Animal_ID) + (1|Animal_ID:Session)

Name	Estimate	SE	tStat	DF	pValue	Lower	Upper
(Intercept)	-0.281074861	0.309393515	-0.908470434	223	0.364610469	-0.890783963	0.328634242
Speed	0.085471799	0.052182895	1.637927497	223	0.102847036	-0.017362891	0.188306488
Genotype_cTKO	-0.046065953	0.382805955	-0.120337609	223	0.904324063	-0.800445939	0.708314032
RestFRClass_LowActive	1.066468577	0.373138227	2.858105922	223	0.004665363	0.331140386	1.801796769
RestFRClass_HighlyActive	0.361595221	0.403962132	0.895121578	223	0.371687245	-0.434476374	1.157666815
Speed:Genotype_cTKO	-0.069464515	0.053708687	-1.293357156	223	0.197225708	-0.175306021	0.036376991
Speed:RestFRClass_LowActive	-0.099118033	0.061698815	-1.606481941	223	0.109583096	-0.220705356	0.022469291
Speed:RestFRClass_HighlyActive	-0.095801088	0.065824842	-1.45539413	223	0.146966456	-0.225519402	0.033917226
Genotype_cTKO:RestFRClass_LowActive	0.105313117	0.523869004	0.201029487	223	0.840858754	-0.92705403	1.137680265
Genotype_cTKO:RestFRClass_HighlyActive	-0.632350957	0.6789108	-0.931419794	223	0.35264372	-1.970252609	0.705550694
Speed:Genotype_cTKO:RestFRClass_LowActive	0.063772413	0.066143911	0.96414638	223	0.336016902	-0.066574679	0.194119505
Speed:Genotype_cTKO:RestFRClass_HighlyActive	0.111285338	0.071266432	1.56153935	223	0.119814545	-0.029156495	0.251727172

Putative Inhibitory Neurons (CA1) *Data at single neuron level from N = 7 control, 4 cTKO animals (one control animal sessile during recordings and insufficient number of interneurons in 2 cTKO mice)*

LME Model: Modulation Index ~ RestFRClass * Genotype * Speed + (1|Animal_ID) + (1|Animal_ID:Session)

Name	Estimate	SE	tStat	DF	pValue	Lower	Upper
(Intercept)	-0.386784344	0.371967783	-1.039832915	49	0.303521387	-1.134281589	0.360712902
Speed	0.046959181	0.061539626	0.763072252	49	0.449078594	-0.076709328	0.17062769
Genotype_cTKO	-0.043330837	0.542864188	-0.079818928	49	0.936706374	-1.134257266	1.047595592
RestFRClass_LowActive	0.605300211	0.484748391	1.248689469	49	0.21771176	-0.368838152	1.579438575
RestFRClass_HighlyActive	0.146113586	0.814822409	0.179319548	49	0.858426254	-1.491333349	1.783560522
Speed:Genotype_cTKO	-0.015709869	0.067423579	-0.233002603	49	0.816729972	-0.151202625	0.119782886
Speed:RestFRClass_LowActive	-0.013545546	0.086548081	-0.156508912	49	0.876274964	-0.187470427	0.160379335
Speed:RestFRClass_HighlyActive	0.0377003	0.125977777	0.299261515	49	0.766006194	-0.21546152	0.290862121
Genotype_cTKO:RestFRClass_LowActive	-2.673656251	5.36455512	-0.498392913	49	0.620436981	-13.45413338	8.106820876
Genotype_cTKO:RestFRClass_HighlyActive	-1.521853077	1.448433632	-1.050688857	49	0.298555906	-4.432589437	1.388883282
Speed:Genotype_cTKO:RestFRClass_LowActive	0.184201677	0.355709808	0.517842557	49	0.606898398	-0.530623945	0.899027299
Speed:Genotype_cTKO:RestFRClass_HighlyActive	0.077647815	0.15353582	0.505730944	49	0.615313161	-0.230893967	0.386189598

Table S4: Awake LFP power differences between genotypes, related to Figure 2H.

Genotype: Control (reference level) or cTKO animals.
Data at animal level from 8 Control and 6 cTKO mice.

LME Model: LFP_Power ~ Genotype + (1| Animal_ID) + (1|Animal_ID:Session)

Cortex (Low frequency <30Hz)

Name	Estimate	SE	tStat	DF	pValue	Lower	Upper
(Intercept)	0.93042383	0.007892474	117.8875	39	2.08445E-51	0.914459794	0.946387864
Genotype_cTKO	-0.02663957	0.012044563	-2.21175	39	0.032911337	-0.051001994	-0.002277137

Cortex (High frequency >30Hz)

Name	Estimate	SE	tStat	DF	pValue	Lower	Upper
(Intercept)	0.06664278	0.007592351	8.777620545	39	8.96966E-11	0.051285799	0.08199976
Genotype_cTKO	0.025522041	0.011586803	2.202681827	39	0.033594112	0.002085519	0.048958563

CA1 (Low frequency <30Hz)

Name	Estimate	SE	tStat	DF	pValue	Lower	Upper
(Intercept)	0.854496222	0.01123541	76.0538547	39	5.13021E-44	0.831770461	0.877221984
Genotype_cTKO	-0.06447347	0.017123388	-3.76522855	39	0.000548695	-0.099108791	-0.029838148

CA1 (High frequency >30Hz)

Name	Estimate	SE	tStat	DF	pValue	Lower	Upper
(Intercept)	0.141134287	0.010690813	13.2014549	39	5.7073E-16	0.11951008	0.162758497
Genotype_cTKO	0.062963179	0.016289752	3.86520185	39	0.000408866	0.03001405	0.095912311

Table S5: Linear mixed effects modelling to examine effect of MK-801 treatment on neuronal activity between genotypes, related to Figure 5B-C.

FR: Firing or calcium transient rate of individual neurons.
Treatment: Baseline (pre-MK-801 treatment) or 45mins post-MK801 injection.
Data at the neuron level from 4 mice

LME Model: FR ~ Treatment + (1|Animal_ID) + (1|Animal_ID:NeuronID)

Cortex (all neurons) – Neuropixels recordings							
Name	Estimate	SE	tStat	DF	pValue	Lower	Upper
(Intercept)	0.691303039	0.10041238	6.88463955	654	1.361E-11	0.4941335	0.888472579
Treatment_MK45	-0.180325547	0.025397083	-7.10024642	654	3.25611E-12	-0.230195206	-0.130455888
**CA1 (all neurons) – Neuropixels recordings**							
Name	Estimate	SE	tStat	DF	pValue	Lower	Upper
(Intercept)	1.45880895	0.203794207	7.15824543	536	2.705E-12	1.0584757	1.85914223
Treatment_MK45	-0.11629806	0.048726931	-2.38673056	536	0.017344782	-0.2120172	-0.02057889
**Layer 2/3 cortex (all neurons) – 2-photon recordings**							
Name	Estimate	SE	tStat	DF	pValue	Lower	Upper
(Intercept)	1.09097307	0.067241489	16.2247013	284	2.4101E-42	0.958618141	1.22333
Treatment_MK45	-1.05390973	0.057569817	-18.3066368	284	5.55639E-50	-1.1672274	-0.94059

Table S6: Linear mixed effects modelling to examine effect of DCS treatment on neuronal activity between genotypes, related to Figure 7B.

NormFR: Firing rate normalised to baseline (pre-DCS treatment).
RestFRClass: Neurons were categorized according to their resting state firing rate: low-activity <0.1Hz and high-activity >4Hz (normoactive in intervening range) corresponding to lower and upper bounds of slow-delta frequency bands.
Genotype: Control (reference level) or cTKO animals.
DCSTime: DCS drug treatment time as a continuous variable (0mins [i.e. baseline pre-DCS treatment], 7mins, 20mins and 45mins).
DCSTimeSquared: Squared DCS time to model non-linearities in response to DCS treatment over time.
Data at the animal level from 4 cTKO and 3 Control mice.

LME Model: NormFR ~ RestFRClass *Genotype * DCSTime + RestFRClass *Genotype * DCSTimeSquared + (1|Animal_ID)

Putative Excitatory Neurons (Cortex)

Name	Estimate	SE	tStat	DF	pValue	Lower	Upper
(Intercept)	1.015039434	0.684150935	1.483648391	66	0.142662284	-0.35091198	2.380990848
Genotype_cTKO	-0.072659689	0.905046617	-0.080282814	66	0.936255271	-1.879643562	1.734324183
RestFRClass_LowActive	0.104151534	0.967535531	0.107646211	66	0.914602909	-1.827595481	2.03589855
RestFRClass_HighlyActive	0.092539908	0.967535531	0.095644971	66	0.924092364	-1.839207108	2.024286923
DCSTime	-0.008164516	0.088529069	-0.09222413	66	0.92679933	-0.184918506	0.168589473
DCSTimeSquared	0.000159504	0.001838019	0.086780283	66	0.931108907	-0.00351022	0.003829228
Genotype_cTKO:RestFRClass_LowActive	-0.078813013	1.2799292	-0.061576073	66	0.951086524	-2.634274113	2.476648086
Genotype_cTKO:RestFRClass_HighlyActive	0.048444341	1.2799292	0.037849235	66	0.969922141	-2.507016758	2.603905441
Genotype_cTKO:DCSTime	0.021527441	0.117112951	0.183817762	66	0.854720007	-0.21229611	0.255350991
RestFRClass_LowActive:DCSTime	0.101419704	0.12519901	0.810067936	66	0.42081232	-0.148548186	0.351387594
RestFRClass_HighlyActive:DCSTime	-0.009002998	0.12519901	-0.071909496	66	0.942891402	-0.258970887	0.240964892
Genotype_cTKO:DCSTimeSquared	-0.000787885	0.002431471	-0.324036232	66	0.746935272	-0.005642473	0.004066704
RestFRClass_LowActive:DCSTimeSquared	-0.001255105	0.002599352	-0.482853036	66	0.63079789	-0.006444879	0.003934669
RestFRClass_HighlyActive:DCSTimeSquared	0.000111946	0.002599352	0.043066952	66	0.965778193	-0.005077827	0.00530172
Genotype_cTKO:RestFRClass_LowActive:DCSTime	0.374652974	0.165622723	2.262086793	66	0.026990749	0.043976539	0.70532941
Genotype_cTKO:RestFRClass_HighlyActive:DCSTime	-0.032420106	0.165622723	-0.195746726	66	0.845409831	-0.363096541	0.29825633
Genotype_cTKO:RestFRClass_LowActive:DCSTimeSquared	-0.007303037	0.003438619	-2.123828363	66	0.037436432	-0.014168463	-0.000437612
Genotype_cTKO:RestFRClass_HighlyActive:DCSTimeSquared	0.00069405	0.003438619	0.201839706	66	0.840662873	-0.006171375	0.007559475

Putative Excitatory Neurons (CA1)

Name	Estimate	SE	tStat	DF	pValue	Lower	Upper
(Intercept)	1.084128857	0.686295492	1.579682322	66	0.118960738	-0.286104304	2.454362017
Genotype_cTKO	-0.141775476	0.907883599	-0.156160411	66	0.876383256	-1.954423567	1.670872614
RestFRClass_LowActive	-0.104516866	0.970568393	-0.107686245	66	0.914571274	-2.042319185	1.833285454
RestFRClass_HighlyActive	-0.137480356	0.970568393	-0.141649323	66	0.887788496	-2.075282675	1.800321963
DCSTime	0.046088066	0.088806575	0.518971327	66	0.605516619	-0.131219981	0.223396113
DCSTimeSquared	-0.000432397	0.001843781	-0.234516393	66	0.815310124	-0.004113624	0.00324883
Genotype_cTKO:RestFRClass_LowActive	-0.044333167	1.283941299	-0.034528967	66	0.97255956	-2.607804681	2.519138346
Genotype_cTKO:RestFRClass_HighlyActive	0.278358768	1.283941299	0.216800229	66	0.829032793	-2.285112746	2.841830281
Genotype_cTKO:DCSTime	-0.076184222	0.117480056	-0.648486426	66	0.518920364	-0.310740721	0.158372278
RestFRClass_LowActive:DCSTime	0.279428033	0.125591462	2.22489672	66	0.029511722	0.028676588	0.530179478
RestFRClass_HighlyActive:DCSTime	-0.054154902	0.125591462	-0.431198909	66	0.667728904	-0.304906347	0.196596544
Genotype_cTKO:DCSTimeSquared	0.000779828	0.002439093	0.319720606	66	0.750190024	-0.004089978	0.005649634
RestFRClass_LowActive:DCSTimeSquared	-0.00447825	0.0026075	-1.717449715	66	0.090586248	-0.009684292	0.000727792
RestFRClass_HighlyActive:DCSTimeSquared	0.000563313	0.0026075	0.216035701	66	0.829626221	-0.004642729	0.005769355
Genotype_cTKO:RestFRClass_LowActive:DCSTime	0.415744735	0.166141888	2.502347475	66	0.014822517	0.084031752	0.747457717
Genotype_cTKO:RestFRClass_HighlyActive:DCSTime	0.058296208	0.166141888	0.350882057	66	0.72679438	-0.273416775	0.39000919
Genotype_cTKO:RestFRClass_LowActive:DCSTimeSquared	-0.008983002	0.003449398	-2.604222875	66	0.01136472	-0.015869947	-0.002096056
Genotype_cTKO:RestFRClass_HighlyActive:DCSTimeSquared	-0.000742682	0.003449398	-0.215307726	66	0.830191368	-0.007629628	0.006144264

Table S7: Linear mixed effects modelling to examine effect of DCS treatment on open-field behavior between genotypes, related to Figure 7F-G.

Genotype: Control (reference level) or cTKO animals.
TreatmentCondition: Saline control (reference level) or DCS drug treatment.
SequenceOrder: Order of treatments in crossover trial; Saline control as first injection ('SalineFirst') or DCS as first injection ('DCSFirst').
Data at the animal level from 6 cTKO and 6 Control mice (each animal received both saline and DCS injections).

LME Model: OF Measurement ~ TreatmentCondition * Genotype * SequenceOrder + (1|Animal_ID)

Locomotion

Name	Estimate	SE	tStat	DF	pValue	Lower	Upper
(Intercept)	67.3742535	53.1903165	1.266663895	16	0.223405052	-45.3841803	180.1326874
Genotype_cTKO	285.4171342	75.222467	3.794307015	16	0.001591737	125.9526278	444.8816406
TreatmentCondition_DCS	59.87621213	71.7636356	0.834353104	16	0.416365933	-92.2558993	212.0083236
SequenceOrder_SalineFirst	91.57632375	75.222467	1.217406546	16	0.241100784	-67.8881827	251.0408302
Genotype_cTKO:TreatmentCondition_DCS	-273.630726	101.489107	-2.69615858	16	0.015896563	-488.778021	-58.4834305
Genotype_cTKO:SequenceOrder_SalineFirst	-257.837494	106.380633	-2.42372589	16	0.027583735	-483.354362	-32.3206268
TreatmentCondition_DCS:SequenceOrder_SalineFirst	-137.575416	101.489107	-1.3555683	16	0.194063788	-352.722711	77.57187908
Genotype_cTKO:TreatmentCondition_DCS:SequenceOrder_SalineFirst	260.4732712	143.527271	1.814799856	16	0.088346707	-43.7909517	564.7374941

Stereotypy

Name	Estimate	SE	tStat	DF	pValue	Lower	Upper
(Intercept)	0.940892684	0.025052314	37.5571169	16	4.94277E-17	0.88778415	0.994001217
Genotype_cTKO	0.146250568	0.035429322	4.127952755	16	0.000788943	0.07114376	0.221357376
TreatmentCondition_DCS	0.099966127	0.025370804	3.94020334	16	0.00117037	0.046182425	0.153749828
SequenceOrder_SalineFirst	0.118214633	0.035429322	3.336632647	16	0.004183547	0.043107825	0.193321441
Genotype_cTKO:TreatmentCondition_DCS	-0.171522042	0.035879735	-4.78047126	16	0.000204305	-0.247583683	-0.095460402
Genotype_cTKO:SequenceOrder_SalineFirst	-0.154889058	0.050104628	-3.09131242	16	0.007006203	-0.261106125	-0.048671992
TreatmentCondition_DCS:SequenceOrder_SalineFirst	-0.219164269	0.035879735	-6.10830231	16	1.51057E-05	-0.295225909	-0.143102628
Genotype_cTKO:TreatmentCondition_DCS:SequenceOrder_SalineFirst	0.272354544	0.050741608	5.367479561	16	6.2864E-05	0.16478714	0.379921947
